# Genetic studies of focal segmental glomerulosclerosis: a waste of scientific time?

**DOI:** 10.1007/s00467-018-4161-6

**Published:** 2018-12-27

**Authors:** Alexander J. Howie

**Affiliations:** Department of Pathology, Department of Cellular Pathology, Royal Free Hospital, University College London, London, NW3 2QG UK

**Keywords:** Focal segmental glomerulosclerosis, Genetics, Renal pathology

## Abstract

Many genetic causes of focal segmental glomerulosclerosis (FSGS) have been described. A paradox is that the science in the molecular biology, which generally appears of high quality, is not mirrored by a similarly critical analysis of the renal pathology. FSGS has been applied to such a wide range of conditions that it can reasonably be said to have no useful meaning. Attempts to refine the term have been largely ignored. Study of 252 papers on genetic causes of FSGS found various clinical features. Many papers took the reported diagnosis without question. Few papers reported a pathological review, almost half reported FSGS and up to six other conditions caused by any particular gene, some reported FSGS with recognisable glomerular disorders, over 80% did not apply the Columbia classification, and in nearly all with photomicrographs, the images were not useful for refinement of FSGS. Some workers commented on a lack of genotype-phenotype correlation. One reason is a disregard of the principle that scientific investigation requires an unambiguous definition of the condition studied, to allow others to replicate or refute the findings. Genetic studies of FSGS should use a similarly rigorous approach to renal pathology to that used in molecular biology.

**Electronic supplementary material:**

The online version of this article (10.1007/s00467-018-4161-6) contains supplementary material, which is available to authorized users.

## Introduction

Many genetic causes of focal segmental glomerulosclerosis (FSGS) have been described. As examples, Gast et al. [1] gave a list of 39 genes associated with FSGS, Preston et al. [2] listed 48, and De Vriese et al. [3] listed 55, separated into renal limited (21) and syndromic (34), although even this last paper did not include some genes mentioned by others, such as Warejko et al. [4]. This means that there are no agreed criteria for identification of genetic causes. Also, sometimes FSGS and steroid-resistant nephrotic syndrome have been regarded as virtually synonymous and not differentiated [5–7]. Other genetic causes of FSGS and/or steroid-resistant nephrotic syndrome continue to be reported, and any paper attempting to show a definitive list of causative genes is out of date before publication.

The molecular biology in papers on genetic causes is presumably rigorously considered by referees and editors before acceptance by journals. Mutation of a single base pair in an exon of a gene is commonly reported as a significant finding, although other genetic features such as polymorphisms are also said to be associated with FSGS. The mere finding of an alteration in a gene is no longer enough by itself to justify the conclusion that the name of the gene can be added to the list of those in which a mutation can cause FSGS. Various conditions should be satisfied before this can be done, listed and discussed in detail by Lovric et al. [8] and others [4, 6]. For example, detailed experimental functional studies are now often required to support the view that any putative new genetic cause of FSGS is indeed likely to be a cause [e.g. 9, 10].

A scientific paradox considered in this review is the discrepancy between the rigour, precision, close control, reproducibility and generally high quality of the molecular biology and if applicable the experimental work in studies of genetic causes of FSGS and the widespread lack of any of these properties in the selection of cases that are included in reports.

## Focal segmental glomerulosclerosis

FSGS continues to be used as a diagnosis even though virtually every paper or review accepts that the term has no useful, definable meaning applicable to a single condition [8, 11–13]. This review concentrates on the almost universal neglect of application of the same scientific principles to selection and diagnosis of cases compared with the application of those scientific principles to the molecular biological methods used to study them. The basis of the review is 252 papers published before September 2018, selected by the criterion that at least one mutation or polymorphism in at least one gene was said to be associated with at least one case of so-called FSGS, whether or not there was any further clarification of that term [Supplement]. With such a rapidly developing topic, this selection cannot be comprehensive, but any papers missed will have little effect on the overall conclusions.

FSGS is often defined tautologously as the finding on microscopy of sclerosed areas, meaning areas that appear solid and stain black with periodic acid-methenamine silver, that are segmental, meaning in only part of a glomerular tuft, and focal, meaning not in every glomerulus. There is a view that more detailed pathological study does not contribute to understanding of FSGS. As examples, comments can be found that ‘At a practical therapeutic level all FSGS should be considered equal’ [14] and ‘The etiopathogenesis of FSGS cannot be reliably determined by light microscopy alone’ [3]. Contrary to this view, detailed pathological investigation has shown that cases labelled FSGS can be separated into various types, depending on features such as the position of lesions within glomeruli, the state of the kidney outside glomeruli and other things. For example, when clinical features are also taken into account, such as the clinical presentation and apparent renal mass, the single term FSGS is seen to be completely inadequate and thoroughly misleading for the range of conditions to which it is applied [15–17].

Even the common attempt at refinement by the division into primary and secondary types of FSGS is crude, imprecise and unsatisfactory. What do these terms mean? Is FSGS caused by a genetic disorder primary or secondary or neither? FSGS of presumed genetic cause is often said to be not primary, and so is presumably or explicitly secondary [11, 13, 18], or is explicitly neither primary nor secondary but separate from these [3], or is considered not secondary, and so is presumably or explicitly primary [6, 19, 20]. Bierzynska and Saleem [6] said that patients with non-nephrotic proteinuria and FSGS but without an underlying cause were considered to have primary FSGS, although these do not have recurrence after transplantation, unlike many patients with the nephrotic syndrome and ‘primary’ FSGS. Primary FSGS associated with the nephrotic syndrome is often thought to be caused by something in the blood, which means that it is secondary to whatever is circulating [6, 12, 20]. This emphasises the inconsistent application of the term primary FSGS. Are not all lesions secondary to some underlying cause? Do primary, ‘secondary’ and genetic forms of FSGS have any connection with each other? If not, what is the use of the term ‘FSGS’?

The Columbia classification of FSGS was an attempt by pathologists to reduce the ambiguity of the term FSGS by the introduction of five types or variants, called cellular, collapsing, hilar, tip and not otherwise specified (NOS) [21]. Even though this classification has flaws, it is better than nothing in its help to nephrologists [17]. For example, the tip variant of FSGS has a better outcome than other variants [20, 22, 23]. Tests of reproducibility have shown that there is agreement between pathologists in application of the Columbia classification [24]. A study of recurrent FSGS in renal allografts showed that four fifths of cases showed the same Columbia variant as the original disease [25].

## Selection of cases in genetic causes of focal segmental glomerulosclerosis

There was a range of ways in which people were identified and reported to have a genetic cause of FSGS. Most were found by study of groups with features in common, but some were explicitly reports of a single case or more than one case. The distinction between a case report and a series was not always easy to make, especially in papers about families with an apparently inherited condition. Of the 252 papers, 199 were regarded as series in the broadest sense, including most with familial cases, and 53 as case reports. Familial cases were reported in 71 papers, sporadic cases in 25 and both familial and sporadic cases in 56, while 100 papers did not say whether cases were familial or sporadic. There was a range of ages studied. Only children were included in 119 papers, only adults in 50 and both children and adults in 74, while nine papers did not specify the age of subjects.

Clinical features of cases varied. There were three reasons why there was difficulty in determining precise numbers of subjects with different clinical features. At least 17 papers included patients reported in previous studies, sometimes without identifying which ones were included. Patients with different features were grouped in some papers. Some papers did not even give clinical details. The nephrotic syndrome was a common clinical finding and a common reason for inclusion in series, but sometimes, this was specifically only steroid-resistant nephrotic syndrome, sometimes both steroid-resistant and steroid-sensitive nephrotic syndrome, sometimes unspecified nephrotic syndrome and sometimes not differentiated from non-nephrotic proteinuria. Congenital nephrotic syndrome was sometimes included with other types of the nephrotic syndrome, sometimes excluded, sometimes the only clinical feature and frequently not mentioned in childhood studies.

For the reasons given, precise numbers of these different features cannot be determined, but at least 145 series appear to have had the nephrotic syndrome as the main or only clinical feature. Other clinical features, if they were mentioned, included such things as non-nephrotic proteinuria, microscopic haematuria, renal failure, hypertension and combinations of features. Some of these were the main reason for inclusion in series.

A diagnosis of FSGS was a reason for selection of cases for study in 70 series, but this was accompanied by a variety of clinical features, and was not necessarily the only reason for selection. Accordingly, there is an overlap between these 70 papers and many with the nephrotic syndrome. Thirteen papers did not specify any clinical features in those with FSGS. Ten papers selected FSGS specifically with the nephrotic syndrome but not all with the same type of nephrotic syndrome, as described above, while 34 papers selected FSGS with the nephrotic syndrome or other clinical features, eight selected either FSGS or the nephrotic syndrome and five selected FSGS with other glomerular disorders, such as IgA nephropathy or thin glomerular basement membrane disease. In some series, particularly on patients with steroid-resistant nephrotic syndrome, some were said to have FSGS even though a renal biopsy was reported to show something else [26], or patients were assumed to have FSGS [27, 28].

In 62 papers, both series and case reports, the reason for inclusion was a variety of syndromes that included renal disease, not necessarily with the nephrotic syndrome. The most numerous syndromes were those with *WT1* mutations, such as Frasier and Denys-Drash syndromes (14 papers), various disorders associated with mitochondrial DNA abnormalities, such as MELAS syndrome and maternally inherited diabetes mellitus and deafness (10), Alport syndrome (6), Charcot-Marie-Tooth syndrome (5), Galloway-Mowat syndrome (3), Pierson syndrome (3) and Schimke immunoosseous dysplasia (3). Among the others were seven with tubular disorders, particularly Dent syndrome (3) and Bartter syndrome (2). Other reasons for inclusion in series were, as examples, that there was a known genetic mutation or polymorphism, or simply that the patients had been referred for genetic testing.

There were various clinical reasons for exclusion of cases from 44 series, particularly causes of secondary FSGS such as reduced renal mass or obesity (28 papers, including 11 with HIV infection) or extrarenal features (8). HIV infection was included in seven other series. The remaining series and case reports either included patients with extrarenal features (59) or did not mention whether there were any exclusions on clinical grounds (142).

In the 252 papers, 91 excluded an abnormality in at least one relevant gene, such as mutations in *NPHS1* and *NPHS2*, a couple of the earliest genes to be described. In theory, every paper should have searched for all reported genetic causes before an apparently new abnormality was described, which may have been a coincidental and irrelevant finding. Similarly, in theory, whenever any new cause is published, authors of every previous paper should review their cases to see whether that cause was missed, and was a more likely cause or contributor to the condition. Bierzynska and Saleem [6] warned that ‘almost all historical reports of causal variants need to be reassessed against current reference datasets, to filter out those that are no longer deemed causative’.

This overview shows that genetic studies of FSGS had no consistency in the population studied. Although many patients with FSGS had steroid-resistant nephrotic syndrome, some did not. Was the FSGS reported in anyone with steroid-resistant nephrotic syndrome, for example, the same as the FSGS reported in a case of congenital nephrotic syndrome, or an adult with asymptomatic proteinuria, or a child with Alport syndrome or someone with a tubular disorder?

The concept of phenocopies of genetic causes of FSGS or steroid-resistant nephrotic syndrome was introduced by Warejko et al. [4], without an explicit definition. This cannot mean disorders which are genetic but do not affect the same cells as ‘genuine’ genetic causes of FSGS, which often seem to be confined to those purely affecting podocytes [8, 29, 30]. For example, one gene said to cause a phenocopy of genetic causes of FSGS was *GLA*, mutated in Fabry disease, which affects podocytes [31]. The non-phenocopy group of Warejko et al. [4] included genes in the syndromic group of De Vries et al. [3], such as *COQ2*, *INF2*, *LAMB2*, *LMX1B*, *SMARCAL1* and *WT1*, and so genes causing FSGS can affect podocytes and cells outside the kidney. Similarly confusing is the finding that several genetic abnormalities that cause tubular disorders [32] have been reported to cause FSGS, such as *CLCN5*, mutated in Dent disease 1 [33], and *SLC12A1*, mutated in Bartter syndrome type 1 [34], although these are not always included in lists of genetic causes of FSGS. Also overlooked sometimes are mitochondrial DNA mutations reported in cases of FSGS [e.g. 35–39], and glycogen storage diseases said to cause FSGS [40, 41].

## The diagnosis of focal segmental glomerulosclerosis in studies

There seemed a readiness of many pathologists to apply the term FSGS and a readiness of those doing the genetic studies to accept the diagnosis. In only 21 papers (8% of 252) was there a mention that there was a review of light microscopic sections on every renal biopsy or every available biopsy or most biopsies by at least one pathologist. This should in theory give consistency in the cases included and excluded [e.g. 38, 42, 43]. Otherwise, the diagnosis of FSGS in series appeared to have been taken from reports by various pathologists. Why this is important is shown by the finding that in nearly half of the papers, 122 out of 252 (48%), any particular gene was reported to cause not only FSGS, but other glomerular abnormalities. This excludes papers mentioning only global sclerosis of glomeruli as another finding. Commonly reported other diagnoses were minimal change nephropathy, mesangial proliferative glomerulonephritis, IgM nephropathy, membranoproliferative glomerulonephritis, diffuse mesangial sclerosis and congenital nephrotic syndrome of Finnish type, but there were many others. As examples, Chernin et al. [44] reported FSGS and four of these other diagnoses in children with *WT1* mutations, and Sadowski et al. [45] reported FSGS and six other diagnoses, including Alport nephropathy, in children with *NPHS2* mutations. Can genetic abnormalities really cause multiple glomerular disorders, or is a more likely explanation that there was inconsistency between pathologists in these studies?

Sometimes, among conditions as well as FSGS that were reported in particular mutations, unsatisfactory terms appeared, such as ‘minimal unspecific glomerular changes’, ‘unclear (or no) diagnosis’, ‘mild (or minor) glomerular changes’, ‘nonspecific tubulointerstitial nephropathy’, ‘not classifiable’, ‘unclassifiable glomerulopathy’, ‘nonspecific pathology’ and ‘no findings’. This again emphasises how unreliable is mere copying of reports from a variety of pathologists, especially those who are not experts in renal pathology. Also, FSGS was said to occur in glomeruli with diagnoses such as IgA nephropathy [46], diabetic glomerulopathy [47], haemolytic-uraemic syndrome [48], Alport syndrome [49] and thin glomerular basement membranes [50]. Did the patients have two disorders, or were the segmental lesions a complication of the other condition? If they were a complication, would the diagnosis be better as, for example, Alport syndrome with sclerosing glomerular lesions, rather than merely FSGS?

Application of the Columbia classification would be a test of whether a paper attempted to refine the diagnosis of FSGS. Columbia appeared in 2004 [21], although it had been suggested by D’Agati in 2003 [51]. Naturally, only papers published after the classification was published could incorporate it in their findings. The year 2005 was arbitrarily selected as the earliest publication date when Columbia, or various related papers, could be quoted [21, 23, 51–53]. Of 200 papers published in 2005 and afterwards, 140 (70%) did not apply or give a reference to Columbia or relevant papers by D’Agati, and 22 (11%) mentioned Columbia/D’Agati but did not apply the classification. Another 24 papers (12%) did not mention Columbia/D’Agati but included at least one term used in Columbia, almost always just collapsing FSGS, a term which had been introduced earlier [54] and so did not necessarily indicate familiarity with Columbia or application of it. Only 14 (7%) papers both mentioned and applied Columbia. Incidentally, the paper that introduced the term collapsing glomerulopathy said that it was ‘clinically, pathologically, and epidemiologically different from noncollapsing FSGS’ [54], although this distinction has often been ignored [17].

Photomicrographs were common in papers on genetic causes of FSGS, and were included in 86 of 252 papers (34%). A problem is that the figures were almost always just of one glomerulus, and an illustration of one glomerulus in itself generally is of little value. For satisfactory interpretation of a segmental glomerular lesion, the ideal information should be a description of the lesion, its site within the glomerulus, which requires the landmarks of the hilum and tubular origin to be shown, the condition and size of the rest of the glomerulus, the number of glomeruli affected and the condition of the rest of the kidney [17]. Hardly any papers satisfied these requirements. In only 12 papers (14% of 86) could the site of a single type of segmental lesion be identified, and this was at the glomerular hilum in 11 of these. In most papers, 48 of 86 (56%), figures showed segmental lesions that either were at unidentifiable sites in glomeruli or were large or at various sites in glomeruli, meaning that they were late lesions [16]. In 17 papers (20%), there were no segmental lesions, either because the figure was indistinct or definitely had no visible lesion or because an abnormality affected the whole glomerulus, which means it was global rather than segmental, and so could not strictly be called FSGS on any definition. Several of these global lesions were said to show collapsing glomerulopathy, which seems to be conventionally or explicitly included in the term FSGS, even though the changes are distinctive and are generally global, not segmental [17, 54]. In the other nine papers (10%), segmental lesions were seen in association with an identifiable disorder, most commonly IgA nephropathy.

No paper mentioned that the position of segmentally sclerosed glomeruli in the cortex was analysed. Following the work of Rich [55], FSGS is often said to affect glomeruli in the inner/juxtamedullary cortex, although this is probably only true in children [17]. Diffuse mesangial sclerosis appears different because this is reported to be worse in the outer cortex [56], but only one paper appears to have investigated this [57]. In virtually all papers, study not only of glomeruli but of the whole kidney tissue in a biopsy sample was unsatisfactory [17].

## Why this is important

These findings on the selection, diagnosis, description and illustration of cases show that the literature on genetic causes of FSGS is in confusion. One reason why this is important is because the first requirement for study of any condition is to have a precise definition of that condition so that as in any scientific study, other workers can investigate and replicate or refute the original findings. At the moment, there can be little certainty that any paper on FSGS is studying the same condition as any other paper. The confused state of genetics of FSGS contrasts sharply with the relatively much more straightforward genetics of membranous nephropathy, a diagnosis that is uncontroversial compared with FSGS [58]. The critical scientific principles applied to the molecular biology do not appear to have been applied to interpretation of the pathological findings of changes called FSGS. A suggested solution for any proposed study is that those working on the genetics do not take a diagnosis of FSGS at face value unless there has been a thorough review of renal biopsy specimens by a pathologist familiar with the latest ideas on segmental sclerosing disorders, and there is an attempt to classify the segmental lesions. Barisoni and others investigated concordance between pathologists in identification of various features in renal biopsy specimens [59]. They suggested that rather than concentration on single features of segmental glomerular sclerosis, which gave inconsistent concordance, better concordance would be produced by use by pathologists of ‘the totality of the histopathology to arrive at a diagnosis’. This has been suggested previously [15–17].

Another reason why this is important is related to understanding of the pathogenesis of segmental lesions. A few papers reporting mitochondrial DNA mutations have shown that the lesions in affected glomeruli are at the vascular pole or hilum and are associated with abnormalities of arterioles [35–37, 39]. Although this does not explain how the lesions develop, it suggests where research could concentrate to find out the pathogenesis. Similarly, a few papers reporting segmental lesions in kidneys with genetic tubular disorders and glycogen storage disorders have indicated that these lesions may be consequences of glomerular functional overload following a reduction in nephron numbers [34, 40, 41].

Hardly any other papers suggested a mechanism of development of segmental sclerosing lesions, other than vague ideas that the lesions were due to podocyte disorders, which would be expected to produce lesions randomly distributed in glomeruli [17]. One paper, not included in the series of 252 papers because it avoided use of the term FSGS as a diagnosis, was Ozaltin et al. [60] on *DGKE* variants causing a glomerular microangiopathy: ‘There were also secondary focal and segmental sclerotic glomeruli, which could be seen in the advanced stage of any glomerulopathy’. Although most genes said to cause FSGS are expressed in podocytes [8, 29, 30], few have suggested how genetic abnormalities could cause segmental sclerosing lesions. A rare example is Henderson et al. [61], who found that perihilar segmental lesions, typical of glomerular overload or hyperperfusion or hyperfiltration or post adaptive changes [17], were common in kidneys with *ACTN4* mutations, even though some biopsies did not seem to show significant loss of renal mass, nor glomerular enlargement. They speculated that podocytes with *ACTN4* mutations were less mechanically robust than normal podocytes, and more sensitive to damage by normal glomerular capillary pressures. No papers analysed the distribution of segmentally sclerosed glomeruli in the cortex to see if genetic disorders showed the typical childhood pattern, with juxtamedullary glomeruli affected first [17, 55].

A more scientific approach to all cases of FSGS may reveal more precise genotype-phenotype correlations than are currently known. Until this is done, the study of genetic causes of FSGS will remain unsatisfactory. Some workers have expressed disillusionment with reported renal biopsy findings. These include Bierzynska et al. [62], who wrote, ‘Biopsy reports did not correlate in any systematic way with genetic results identified’, and Schultheiss et al. [63], whose paper was entitled ‘No evidence for genotype/phenotype correlation in *NPHS1* and *NPHS2* mutations’. The importance of phenotyping in genetic studies, including the renal biopsy diagnosis, has been stressed by others [45, 64]. There is the possibility that there is no correlation between any particular genetic abnormality and the glomerular changes it produces, but until there is a rigorous test of this, no one will know. A more critical approach to FSGS is necessary to reduce the confusion about this ‘overused and misunderstood descriptive phrase’ [65].

## Electronic supplementary material

ESM 1 (DOCX 83 kb)
