# Supplementary material for: Genetic studies of focal segmental glomerulosclerosis: a waste of scientific time?
Source: Pediatr Nephrol. 2018 Dec 27;35(1):9–16. doi: 10.1007/s00467-018-4161-6 (PMC6901409; doi:10.1007/s00467-018-4161-6)
Supplement: Supplementary file 1 — (DOCX 83 kb) [file 467_2018_4161_MOESM1_ESM.docx]

**Supplement**

**Papers included in**

**Genetic studies of focal segmental glomerulosclerosis: a waste of scientific time?**

**Professor A J Howie**

Letters in square brackets after references: c, Either give a reference to the Columbia classification and use it, or use variants in the classification without a reference (papers before 2005 are overlooked); f, Have at least one light microscopic figure illustrating a glomerulus; o, Give at least one other finding in glomeruli in addition to focal segmental glomerulosclerosis apparently caused by a genetic disorder; r, Mention a review of some or all available renal biopsies specifically for the paper.

1. Abid A, Khaliq S, Shahid S, Lanewala A, Mubarak M, Hashmi S, Kazi J, Masood T, Hafeez F, Naqvi SAA, Rizvi SAH, Mehdi SQ (2012) A spectrum of novel *NPHS1* and *NPHS2* gene mutations in pediatric nephrotic syndrome patients from Pakistan. Gene 502: 133-137 [o]
2. Adam J, Connor TMF, Wood K, Lewis D, Naik R, Gale DP, Sayer JA (2014) Genetic testing can resolve diagnostic confusion in Alport syndrome. Clin Kidney J 7: 197-200 [o]
3. Agarwal AK, Zhou XJ, Hall RK, Nicholls K, Bankier A, Van Esch H, Fryns JP, Garg A (2006) Focal segmental glomerulosclerosis in patients with mandibuloacral dysplasia owing to ZMPSTE24 deficiency. J Invest Med 2006; 54: 208-213 [c, f]
4. Akilesh S, Suleiman H, Yu H, Stander MC, Lavin P, Gbadegesin R, Antignac C, Pollak M, Kopp JB, Winn MP, Shaw AS (2011) Arhgap24 inactivates Rac1 in mouse podocytes, and a mutant form is associated with familial focal segmental glomerulosclerosis. J Clin Invest 121: 4127-4137
5. Al-Eisa A, Haider MZ, Srivastva BS (2001) Angiotensin converting enzyme gene insertion / deletion polymorphism in idiopathic nephrotic syndrome in Kuwaiti Arab children. Scand J Urol Nephrol 2001; 35: 239-242 [o]
6. Al-Hamed MH, Al-Sabban E, Al-Mojalli H, Al-Harbi N, Faqeih E, Al Shaya H, Alhasan K, Al-Hissi S, Rajab M, Edwards N, Al-Abbad A, Al-Hassoun I, Sayer JA, Meyer BF (2013) A molecular genetic analysis of childhood nephrotic syndrome in a cohort of Saudi Arabian families. J Hum Genet 58: 480-489 [o]
7. Al-Romaih KI, Genovese G, Al-Mojalli H, Al-Othman S, Al-Manea H, Al-Suleiman M, Al-Jondubi M, Atallah N, Al-Rodayyan M, Weins A, Pollak MR, Adra CN (2011) Genetic diagnosis in consanguineous families with kidney disease by homozygosity mapping coupled with whole-exome sequencing. Am J Kidney Dis 58: 186-195 [f]
8. Anyaegbu EI, Shaw AS, Hruska KA, Jain S (2015) Clinical phenotype of APOL1 nephropathy in young relatives of patients with end-stage renal disease. Pediatr Nephrol 30: 983-989
9. Ardiles LG, Carrasco AE, Carpio JD, Mezzano SA (2005) Late onset of familial nephrotic syndrome associated with a compound heterozygous mutation of the podocin-encoding gene. Nephrology 10: 553-556 [f]
10. Ashraf A, Gee HY, Woerner S, Xie LX, Vega-Warner V, Lovric S, Fang H, Song X, Cattran DC, Avila-Casado C, Paterson AD, Nitschké P, Bole-Feysot C, Cochat P, Esteve-Rudd J, Haberberger B, Allen SJ, Zhou W, Airik R, Otto EA, Barua M, Al-Hamed MH, Kari JA, Evans J, Bierzynska A, Saleem MA, Böckenhauer D, Kleta R, El Desoky S, Hacihamdioglu DO, Gok F, Washburn J, Wiggins RC, Choi M, Lifton RP, Levy S, Han Z, Salviati L, Prokisch H, Williams DS, Pollak M, Clarke CF, Pei Y, Antignac C, Hildebrandt F (2013) *ADCK4* mutations promote steroid-resistant nephrotic syndrome through CoQ10 biosynthesis disruption J Clin Invest 123: 5179-5189 [c, f]
11. Aucella F, Bisceglia L, De Bonis P, Gigante M, Caridi G, Barbano G, Mattioli G, Perfumo F, Gesualdo L, Ghiggeri GM (2006) *WT1* mutations in nephrotic syndrome revisited. High prevalence in young girls, associations and renal phenotypes. Pediatr Nephrol 21: 1393-1398 [o]
12. Bantis C, Heering PJ, Stangou M, Kouri NM, Schwandt C, Memmos D, Rump LC, Ivens K (2011) Influence of aldosterone synthase gene C-344T polymorphism on focal segmental glomerulosclerosis. Nephrology 16: 730-735 [r]
13. Barbaux S, Niaudet P, Gubler MC, Grunfeld JP, Jaubert F, Kuttenn F, Fekete CN, Souleyreau-Therville N, Thibaud E, Fellous M, McElreavey K (1997) Donor splice-site mutations in *WT1* are responsible for Frasier syndrome. Nature Genet 17: 467-470 [o]
14. Barbosa AS, Hadjiathanasiou CG, Theodoridis C, Papathanasiou A, Tar A, Merksz M, Gyorvari B, Sultan C, Dumas R, Jaubert F, Niaudet P, Moreira-Filho CA, Cotinot C, Fellous M (1999) The same mutation affecting the splicing of *WT1* gene is present on Frasier syndrome patients with or without Wilms’ tumor. Hum Mutat 13: 146-153 [o]
15. Barua M, Brown EJ, Charoonratana VT, Genovese G, Sun H, Pollak MR (2013) Mutations in the *INF2* gene account for a significant proportion of familial but not sporadic focal and segmental glomerulosclerosis. Kidney Internat 83: 316-322 [c, o]
16. Barua M, Shieh E, Schlondorff J, Genovese G, Kaplan BS, Pollak MR (2014) Exome sequencing and in vitro studies identified podocalyxin as a candidate gene for focal and segmental glomerulosclerosis. Kidney Internat 85: 124-133 [f]
17. Barua M, Stellacci E, Stella L, Weins A, Genovese G, Muto V, Caputo V, Toka HR, Charoonratana VT, Tartaglia M, Pollak MR (2014) Mutations in *PAX2* associate with adult-onset FSGS. J Am Soc Nephrol 25: 1942-1953 [f, o]
18. Becker-Cohen R, Bruschi M, Rinat C, Feinstein S, Zennaro C, Ghiggeri GM, Frishberg Y (2007) Recurrent nephrotic syndrome in homozygous truncating *NPHS2* mutation is not due to anti-podocin antibodies. Am J Transplant 7: 256-260
19. Becknell B, Zender GA, Houston R, Baker PB, Kim L. McBride KL, Luo W, Hains DS, Borza DB, Schwaderer AL (2011) Novel X-linked glomerulopathy is associated with a *COL4A5* missense mutation in a non-collagenous interruption. Kidney Internat 79: 120-127 [r, f, o]
20. Benetti E, Caridi G, Malaventura C, Dagnino M, Leonardi E, Artifoni L, Ghiggeri GM, Tosatto SCE, Murer L (2010) A novel *WT1* gene mutation in a three-generation family with progressive isolated focal segmental glomerulosclerosis. Clin J Am Soc Nephrol 5: 698-702 [f]
21. Berdeli A, Mir M, Ozkayin N, Serdaroglu E, Tabel Y, Cura A (2005) Association of macrophage migration inhibitory factor -173C allele polymorphism with steroid resistance in children with nephrotic syndrome. Pediatr Nephrol 20: 1566-1571 [o]
22. Berdeli A, Mir S, Yavascan O, Serdaroglu E, Bak M, Aksu N, Oner A, Anarat A, Donmez O, Yildiz N, Sever L, Tabel Y, Dusunsel R, Sonmez F, Cakar N (2007) *NPHS2* (podocin) mutations in Turkish children with idiopathic nephrotic syndrome. Pediatr Nephrol 22: 2031-2040 [o]
23. Berkovic SF, Dibbens LM, Oshlack A, Silver JD, Katerelos M, Vears DF, Lullmann-Rauch R, Blanz J, Zhang KW, Stankovich J, Kalnins RM, Dowling JP, Andermann E, Andermann F, Faldini E, D’Hooge R, Vadlamudi L, Macdonell RA, Hodgson BL, Bayly MA, Savige J, Mulley JC, Smyth GK, Power DA, Saftig P, Bahlo M (2008) Array-based gene discovery with three unrelated subjects shows SCARB2/LIMP-2 deficiency causes myoclonus epilepsy and glomerulosclerosis. Am J Hum Genet 82: 673-684 [c, f]
24. Bertelli R, Ginevri F, Caridi G, Dagnino M, Sandrini S,Di Duca M, Emma F, Sanna-Cherchi S, Scolari F, Neri TM, Murer L, Massella L, Basile G, Rizzoni G, Perfumo F, Ghiggeri GM (2003) Recurrence of focal segmental glomerulosclerosis after renal transplantation in patients with mutations of podocin. Am J Kidney Dis 41: 1314-1321 [f, o]
25. Bettinelli A, Borsa N, Syren ML, Mattiello C, Coviello D, Edefonti A, Giani M, Travi M, Tedeschi S (2005) Simultaneous mutations in the *CLCNKB* and *SLC12A3* genes in two siblings with phenotypic heterogeneity in classic Bartter syndrome. Pediatr Res 58: 1269-1273 [f]
26. Bierzynska A, McCarthy HJ, Soderquest K, Sen ES, Colby E, Ding WY, Nabhan MM, Kerecuk L, Hegde S, Hughes D, Marks S, Feather S, Jones C, Webb NJA, Ognjanovic M, Christian M, Gilbert RD, Sinha MD, Lord GM, Simpson M, Koziell AB, Welsh GI, Saleem MA (2017) Genomic and clinical profiling of a national nephrotic syndrome cohort advocates a precision medicine approach to disease management. Kidney Internat 91: 937-947 [o]
27. Billing H, Muller D, Ruf R, Lichtenberger L, Hildebrandt F, August C, Querfeld U, Haffner D (2004) *NPHS2* mutation associated with recurrence of proteinuria after transplantation. Pediatr Nephrol 19: 561-564 [f]
28. Biyikli NK, Alpay H, Yildiz N, Agachan B, Ergen A, Zeybek U, Bozkurt N, Ispir T (2006) Paraoxonase 1 192 and 55 polymorphisms in nephrotic children. Pediatr Nephrol 21: 649-654
29. Boyer O, Benoit G, Gribouval O, Nevo F, Pawtowski A, Bilge I, Bircan Z, Deschenes G, Guay-Woodford LM, Hall M, Macher MA, Soulami K, Stefanidis CJ, Weiss R, Loirat C, Gubler MC, Antignac C (2010) Mutational analysis of the *PLCE1* gene in steroid resistant nephrotic syndrome. J Med Genet 47: 445-452 [o]
30. Boyer O, Benoit G, Gribouval O, Nevo F, Tete MJ, Dantal J, Gilbert-Dussardier B, Touchard G, Karras A, Presne C, Grunfeld JP, Legendre C, Joly D, Philippe Rieu P, Mohsin N, Hannedouche T, Moal V, Gubler MC, Broutin I, Mollet G, Antignac C (2011) Mutations in *INF2* are a major cause of autosomal dominant focal segmental glomerulosclerosis. J Am Soc Nephrol 2011; 22: 239-245
31. Boyer O, Nevo F, Plaisier E, Funalot B, Gribouval O, Benoit G, Cong EH, Arrondel C, Tete MJ, Montjean R, Richard L, Karras A, Pouteil-Noble C, Balafrej L, Bonnardeaux A, Canaud G, Charasse C, Dantal J, Deschenes G, Deteix P, Dubourg O, Petiot P, Pouthier D, Leguern E, Guiochon-Mantel A, Broutin I, Gubler MC, Saunier S, Ronco P, Vallat JM, Alonso MA, Antignac C, Mollet G (2011) *INF2* mutations in Charcot-Marie-Tooth disease with glomerulopathy. N Engl J Med 365: 2377-2388 [c, f]
32. Boyer O, Woerner S, Yang F, Oakeley EJ, Bolan Linghu B, Gribouval O, Tete MJ, Duca JS, Klickstein L, Damask AJ, Szustakowski JD, Heibel F, Matignon M, Baudouin V, Chantrel F, Champigneulle J, Martin L, Nitschke P, Gubler MC, Johnson KJ, Chibout SD, Antignac C (2013) *LMX1B* mutations cause hereditary FSGS without extrarenal involvement. J Am Soc Nephrol 2013; 24: 1216-1222 [c, f, o]
33. Braun DA, Rao J, Mollet G, Schapiro D, Daugeron MC, Tan W, Gribouval O, Boyer O, Revy P, Jobst-Schwan T, Schmidt JM, Lawson JA, Schanze D, Ashraf S, Ullmann JFP, Hoogstraten CA, Boddaert N, Collinet B, Martin G, Liger D, Lovric S, Furlano M, Guerrera IC, Sanchez-Ferras O, Hu JF, Boschat AC, Sanquer S, Menten B, Vergult S, De Rocker N, Airik M, Hermle T, Shril S, Widmeier E, Gee HY, Choi WI, Sadowski CE, Pabst WL, Warejko JK, Daga A, Basta T, Matejas V, Scharmann K, Kienast SD, Behnam B, Beeson B, Begtrup A, Bruce M, Ch’ng GS, Lin SP, Chang JH, Chen CH, Cho MT, Gaffney PM, Gipson PE, Hsu CH, Kari JA, Ke YY, Kiraly-Borri C, Lai W, Lemyre E, Littlejohn RO, Masri A, Moghtaderi M, Nakamura K, Ozaltin F, Praet M, Prasad C, Prytula A, Roeder ER, Rump P, Schnur RE, Shiihara T, Sinha MD, Soliman NA, Soulami K, Sweetser DA, Tsai WH, Tsai JD, Topaloglu R, Vester U, Viskochil DH, Vatanavicharn N, Waxler JL, Wierenga KJ, Wolf MTF, Wong SN, Leidel SA, Truglio G, Dedon PC, Poduri A, Mane S, Lifton RP, Bouchard M, Kannu P, Chitayat D, Magen D, Callewaert B, van Tilbeurgh H, Zenker M, Antignac A, Hildebrandt F (2017) Mutations in KEOPS-complex genes cause nephrotic syndrome with primary microcephaly. Nature Genet 49: 1529-1538 [c, f, o]
34. Braun DA, Sadowski CE, Kohl S, Lovric S, Astrinidis SA, Pabst WL, Gee HY, Ashraf S, Lawson JA, Shril S, Airik M, Tan W, Schapiro D, Rao J, Choi WI, Hermle T, Kemper MJ, Pohl M, Ozaltin F, Konrad M, Bogdanovic R, Büscher R, Helmchen U, Serdaroglu E, Lifton RP, Antonin W, Hildebrandt F (2016) Mutations in nuclear pore genes *NUP93*, *NUP205* and *XPO5* cause steroid-resistant nephrotic syndrome. Nature Genet 48: 457-465 [f, o]
35. Brown EJ, Schlondorff JS, Becker DJ, Tsukaguchi H, Tonna SJ, Uscinski AL, Higgs HN, Henderson JM5, Pollak MR (2010) Mutations in the formin gene *INF2* cause focal segmental glomerulosclerosis. Nature Genet 42: 72-76 [r, c, f]
36. Bullich G, Domingo-Gallego A, Vargas I, Ruiz P, Lorente-Grandoso L, Furlano M, Fraga G, Madrid A, Ariceta G, Borregan M, Pinero-Fernandez JA, Rodrıguez-Pena L, Ballesta-Martınez MJ, Llano-Rivas I, Menica MA, Balların J, Torrents D, Torra R, Ars E (2018) A kidney-disease gene panel allows a comprehensive genetic diagnosis of cystic and glomerular inherited kidney diseases. Kidney Internat 94: 363-371 [o]
37. Bullich G, Trujillano D, Santín S, Ossowski S, Mendizábal S, Fraga G, Madrid A, Ariceta G, Ballarín J, Torra R, Estivill X, Ars E (2015) Targeted next-generation sequencing in steroid resistant nephrotic syndrome: mutations in multiple glomerular genes may influence disease severity. Eur J Hum Genet 23: 1192-1199 [o]
38. Bullich G, Vargas I, Trujillano D, Mendizábal S, Pinero-Fernandez JA, Fraga G, Garcia-Solano J, Ballarín J, Estivill X, Torra R, Ars E (2017) Contribution of the *TTC21B* gene to glomerular and cystic kidney diseases. Nephrol Dial Transplant 32: 151-156 [c, f, o]
39. Buscher AK, Konrad M, Nagel M, Witzke O, Kribben A, Hoyer PF, Weber S (2012) [Mutations in podocyte genes are a rare cause of primary FSGS associated with ESRD in adult patients.](http://ovidsp.tx.ovid.com/sp-3.7.1b/ovidweb.cgi?&S=OMIGFPMGKCDDLEAPNCPKOEJCKAJLAA00&Complete+Reference=S.sh.21%7c8%7c1) Clin Nephrol 78: 47-53
40. Buscher AK, Kranz B, Buscher R, Hildebrandt F, Dworniczak B, Pennekamp P, Kuwertz-Broking E, Wingen AM, John U, Kemper M, Monnens L, Hoyer PF, Weber S, Konrad M (2010) Immunosuppression and renal outcome in congenital and pediatric steroid-resistant nephrotic syndrome. Clin J Am Soc Nephrol 5: 2075-2084 [o]
41. Buzza M, Wang YY, Dagher H, Babon JJ, Cotton RG, Powell H, Dowling J, Savige J (2001) *COL4A4* mutation in thin basement membrane disease previously described in Alport syndrome. Kidney Internat 60: 480-483 [o]
42. Caridi G, Bertelli R, Carrea A, Di Duca M, Catarsi P, Artero M, Carraro M, Zennaro C, Candiano G, Musante L, Seri M, Ginevri F, Perfumo F, Ghiggeri GM (2001) Prevalence, genetics, and clinical features of patients carrying podocin mutations in steroid-resistant nonfamilial focal segmental glomerulosclerosis. J Am Soc Nephrol 12: 2742-2746
43. Caridi G, Bertelli R, Di Duca M, Dagnino M, Emma F, Muda AO, Scolari F, Miglietti N, Mazzucco G, Murer L, Carrea A, Massella L, Rizzoni G, Perfumo F, Ghiggeri GM (2003) Broadening the spectrum of diseases related to podocin mutations. J Am Soc Nephrol 14: 1278-1286 [o]
44. Caridi G, Bertelli R, Scolari F, Sanna-Cherchi S, Di Duca M, Ghiggeri GM (2003) Podocin mutations in sporadic focal-segmental glomerulosclerosis occurring in adulthood. Kidney Internat 64: 365
45. Caridi G, Gigante M, Ravani P, Trivelli A, Barbano G, Scolari F, Dagnino M, Murer L, Murtas C, Edefonti A, Allegri L, Amore A, Coppo R, Emma F, De Palo T, Penza R, Gesualdo L, Ghiggeri GM (2009) Clinical features and long-term outcome of nephrotic syndrome associated with heterozygous *NPHS1* and *NPHS2* mutations. Clin J Am Soc Nephrol 4: 1065-1072 [o]
46. Caridi G, Lugani F, Dagnino M, Gigante M, Iolascon A, Falco M, Graziano C, Benetti E, Dugo M, Del Prete D, Granata A, Borracelli D, Moggia E, Quaglia M, Rinaldi R, Gesualdo L, Ghiggeri GM (2014) Novel *INF2* mutations in an Italian cohort of patients with focal segmental glomerulosclerosis, renal failure and Charcot-Marie-Tooth neuropathy. Nephrol Dial Transplant 29: suppl 4: 80-86
47. Challis RC, Ring T, Xu Y, Wong EKS, Flossmann O, Roberts ISD, Ahmed S, Wetherall M, Salkus G, Brocklebank V, Fester J, Strain L, Wilson V, Wood KM, Marchbank KJ, Santibanez-Koref M, Goodship THJ, Kavanagh D (2017) Thrombotic microangiopathy in inverted formin 2-mediated renal disease. J Am Soc Nephrol 28: 1084-1091 [f, o]
48. Chatterjee R, Hoffman M, Cliften P, Seshan S, Liapis H, Jain S (2013) Targeted exome sequencing integrated with clinicopathological information reveals novel and rare mutations in atypical, suspected and unknown cases of Alport syndrome or proteinuria. PLOS ONE 8: e76360
49. Cheong HI, Chae JH, Kim JS, Park HW, Ha IS, Hwang YS, Lee HS, Choi Y (1999) Hereditary glomerulopathy associated with a mitochondrial tRNA^Leu^ gene mutation. Pediatr Nephrol 13: 477-480
50. Chernin G, Heeringa SF, Vega-Warner V, Schoeb DS, Nurnberg P, Hildebrandt F (2010) Adequate use of allele frequencies in Hispanics *-* a problem elucidated in nephrotic syndrome. Pediatr Nephrol 25: 261-266 [o]
51. Chernin G, Vega-Warner V, Schoeb DS, Heeringa SF, Ovunc B, Saisawat P, Cleper R, Ozaltin F, Hildebrandt F (2010) Genotype/phenotype correlation in nephrotic syndrome caused by *WT1* mutations. Clin J Am Soc Nephrol 5: 1655-1662 [o]
52. Cho HY, Lee JH, Choi HJ, Lee BH, Ha IS, Choi Y, Cheong HI (2008) *WT1* and *NPHS2* mutations in Korean children with steroid-resistant nephrotic syndrome. Pediatr Nephrol 23: 63-70 [o]
53. Choi HJ, Lee BH, Cho HY, Moon KC, Ha IS, Nagata M, Choi Y, Cheong HI (2008) Familial focal segmental glomerulosclerosis associated with an *ACTN4* mutation and paternal germline mosaicism. Am J Kidney Dis 51: 834-838 [c, f]
54. Choi HJ, Lee BH, Kang JH, Jeong HJ, Moon KC, Ha IS, Yu YS, Matejas V, Zenker M, Choi Y, Cheong HI (2008) Variable phenotype of Pierson syndrome. Pediatr Nephrol 23: 995-1000
55. Clewing JM, Fryssira H, Goodman D, Smithson SF, Sloan EA, Lou S, Huang Y, Choi K, Lucke T, Alpay H, Andre JL, Asakura Y, Biebuyck-Gouge N, Bogdanovic R, Bonneau D, Cancrini C, Cochat P, Cockfield S, Collard L, Cordeiro I, Cormier-Daire V, Cransberg K, Cutka K, Deschenes G, Ehrich JHH, Frund S, Georgaki H, Guillen-Navarro E, Hinkelmann B, Kanariou M, Kasap B, Sebnem Kilic S, Lama G, Lamfers P, Loirat C, Majore S, Milford D, Morin DC, Ozdemir N, Pontz BF, Proesmans W, Psoni S, Reichenbach H, Reif S, Rusu C, Saraiva JM, Sakallioglu O, Schmidt B, Shoemaker L, Sigaudy S, Smith G, Sotsiou F, Stajic N, Stein A, Stray-Pedersen A, Taha D, Taque S, Tizard J, Tsimaratos M, Wong NACS, Boerkoel CF (2007) Schimke immunoosseous dysplasia: suggestions of genetic diversity. Hum Mutat 28: 273-283
56. Colin E, Cong EH, Mollet M, Guichet A, Gribouval O, Arrondel C, Boyer O, Daniel L, Gubler MC, Ekinci Z, Tsimaratos M, Chabrol B, Boddaert N, Verloes A, Chevrollier A, Gueguen N, Desquiret-Dumas V, Ferre M, Procaccio V, Richard L, Funalot B, Moncla A, Bonneau D, Antignac C (2014) Loss-of-function mutations in *WDR73* are responsible for microcephaly and steroid-resistant nephrotic syndrome: Galloway-Mowat syndrome. Am J Hum Genet 95: 637-648 [c, f]
57. Cong EH, Bizet AA, Boyer O, Woerner S, Gribouval O, Filhol E, Arrondel C, Thomas S, Silbermann F, Canaud G, Hachicha J, Ben Dhia N, Peraldi MN, Harzallah K, Iftene D, Daniel L, Willems M, Noel LH, Bole-Feysot C, Nitschké P, Gubler MC, Mollet G, Saunier S, Antignac C (2014) A homozygous missense mutation in the ciliary gene *TTC21B* causes familial FSGS. J Am Soc Nephrol 25: 2435-2443 [r, f, o]
58. Copelovitch L, Nash MA, Kaplan BS (2007) Hypothesis: Dent disease is an underrecognized cause of focal glomerulosclerosis. Clin J Am Soc Nephrol 2: 914-918 [c]
59. Dai S, Wang Z, Pan X, Chen X, Wang W, Ren H, Feng Q, He JC, Han B, Chen N (2009) *ACTN4* gene mutations and single nucleotide polymorphisms in idiopathic focal segmental glomerulosclerosis. Nephron Clin Pract 111: c87-c94
60. Dai S, Wang Z, Pan X, Wang W, Chen X, Ren H, Hao C, Han B, Chen N (2010) Functional analysis of promoter mutations in the *ACTN4* and *SYNPO* genes in focal segmental glomerulosclerosis. Nephrol Dial Transplant 25: 824-835
61. Demmer L, Primack W, Loik V, Brown R, Therville N, McElreavey K (1999) Frasier syndrome: a cause of focal segmental glomerulosclerosis in a 46,XX female. J Am Soc Nephrol 10: 2215-2218 [f]
62. Denamur E, Bocquet N, Baudouin V, Da Silva F, Veitia R, Peuchmaur M, Elion J, Gubler MC, Fellous M, Niaudet P, Loirat C (2000) *WT1* splice-site mutations are rarely associated with primary steroid-resistant focal and segmental glomerulosclerosis. Kidney Internat 57: 1868-1872 [f, o]
63. Denamur E, Bocquet N, Mougenot B, Da Silva F, Martinat L, Loirat C, Elion J, Bensman A, Ronco PM (1999) Mother-to-child transmitted *WT1* splice-site mutation is responsible for distinct glomerular diseases. J Am Soc Nephrol 10: 2219-2223 [f, o]
64. Ding WY, Koziell A, McCarthy HJ, Bierzynska A, Bhagavatula MK, Dudley JA, Inward CD, Coward RJ, Tizard J, Reid C, Antignac C, Boyer O, Saleem MA (2014) Initial steroid sensitivity in children with steroid-resistant nephrotic syndrome predicts post-transplant recurrence. J Am Soc Nephrol 25: 1342-1348
65. Diomedi-Camassei F, Di Giandomenico S, Santorelli FM, Caridi G, Piemonte F, Montini G, Ghiggeri GM, Murer L, Barisoni L, Pastore A, Muda AO, Valente ML, Bertini E, Emma F (2007) *COQ2* nephropathy: a newly described inherited mitochondriopathy with primary renal involvement. J Am Soc Nephrol 18: 2773-2780 [c, f, o]
66. Ebarasi L, Ashraf S, Bierzynska A, Gee HY, McCarthy HJ. Lovric S, Sadowski CE, Pabst W, Vega-Warner V, Fang H, Koziell A, Simpson MA, Dursun I, Serdaroglu E, Levy S, Saleem MA, Hildebrandt F, Majumdar A (2015) Defects of CRB2 cause steroid-resistant nephrotic syndrome. Am J Hum Genet 96: 153-161
67. Esposito T, Lea RA, Maher BH, Moses D, Cox HC, Magliocca S, Angius A, Nyholt DR, Titus T, Kay T, Gray NA, Rastaldi MP, Parnham A, Gianfrancesco F, Griffiths LR (2013) Unique X-linked familial FSGS with co-segregating heart block disorder is associated with a mutation in the *NXF5* gene. Hum Molec Genet 22: 3654-3666 [f, o]
68. Feltran LS, Varela P, Silva ED, Veronez CL, Franco MC, Filho AP, Camargo MF, Nogueira PCK, Pesquero JB (2017) Targeted next-generation sequencing in Brazilian children with nephrotic syndrome submitted to renal transplant. Transplantation 101: 2905-2912 [o]
69. Fine DM, Wasser WG, Estrella MM, Atta MG, Kuperman M, Shemer R, Rajasekaran A, Tzur S, Racusen LC, Skorecki K (2012) *APOL1* risk variants predict histopathology and progression to ESRD in HIV-related kidney disease. J Am Soc Nephrol 23: 343-350 [r, o]
70. Freedman BI, Hicks PJ, Bostrom MA, Cunningham ME, Liu Y, Divers J, Kopp JB, Winkler CA, Nelson GW, Langefeld CD, Bowden DW (2009) Polymorphisms in the non-muscle myosin heavy chain 9 gene (*MYH9*) are strongly associated with end-stage renal disease historically attributed to hypertension in African Americans. Kidney Internat 75: 736-745 [o]
71. Freedman BI, Nagaraj SK, Lin JJ, Gautreaux MD, Bowden DW, Iskandar SS, Stratta RJ, Rogers J, Hartmann EL, Farney AC, Reeves-Daniel AM (2009) Potential donor-recipient MYH9 genotype interactions in posttransplant nephrotic syndrome after pediatric kidney transplantation. Am J Transplant 9: 2435-2440 [c, f]
72. Frishberg Y, Dinour D, Belostotsky R, Becker-Cohen R, Rinat C, Feinstein S, Navon-Elkan P, Ben-Shalom E (2009) Dent’s disease manifesting as focal glomerulosclerosis: is it the tip of the iceberg? Pediatr Nephrol 24: 2369-2373 [c, o]
73. Frishberg Y, Rinat C, Megged O, Shapira E, Feinstein S, Raas-Rothschild A (2002) Mutations in *NPHS2* encoding podocin are a prevalent cause of steroid-resistant nephrotic syndrome among Israeli-Arab children. J Am Soc Nephrol 13: 400-405 [o]
74. Frishberg Y, Toledano H, Becker-Cohen R, Feigin E, MD, Halle D (2000) Genetic polymorphism in paraoxonase is a risk factor for childhood focal segmental glomerulosclerosis. Am J Kidney Dis 36: 1253-1261
75. Gasser DL, Winkler CA, Peng M, An P, McKenzie LM, Kirk GD, Shi Y, Xie LX, Marbois BN, Clarke CF, Kopp JB (2013) Focal segmental glomerulosclerosis is associated with a *PDSS2* haplotype and, independently, with a decreased content of coenzyme Q10. Am J Physiol Renal Physiol 305: F1228-F1238 [c]
76. Gast C, Pengelly RJ, Lyon M, Bunyan DJ, Seaby EG, Graham N, Venkat-Raman G, Ennis S (2016) Collagen (*COL4A*) mutations are the most frequent mutations underlying adult focal segmental glomerulosclerosis. Nephrol Dial Transplant 31: 961-970 [o]
77. Gbadegesin RA, Hall G, Adeyemo A, Hanke N, Tossidou I, James Burchette J, Wu G, Homstad A, Sparks MA, Gomez J, Jiang R, Alonso A, Lavin P, Conlon P, Korstanje R, Stander MC, Shamsan G, Barua M, Spurney R, Singhal PC, Kopp JB, Haller H, Howell D, Pollak MR, Shaw AS, Schiffer M, Winn MP (2014) Mutations in the gene that encodes the F-actin binding protein anillin cause FSGS. J Am Soc Nephrol 25: 1991-2002 [f]
78. Gbadegesin RA, Lavin PJ, Hall G, Bartkowiak B, Homstad A, Jiang R, Wu G, Byrd A, Lynn K, Wolfish N, Ottati C, Stevens P, Howell D, Conlon P, Winn MP (2012) Inverted formin 2 mutations with variable expression in patients with sporadic and hereditary focal and segmental glomerulosclerosis. Kidney Internat 81: 94-99 [r, f]
79. Gee HY, Zhang F, Ashraf S, Kohl S, Sadowski CE, Vega-Warner V, Zhou W, Lovric S, Fang H, Nettleton M, Zhu J, Hoefele J, Weber LT, Podracka L, Boor A, Fehrenbach H, Innis JW, Washburn J, Levy S, Lifton RP, Otto EA, Han Z, Hildebrandt F (2015) *KANK* deficiency leads to podocyte dysfunction and nephrotic syndrome. J Clin Invest 125: 2375-2384
80. Gellermann J, Stefanidis CJ, Mitsioni A, Querfeld U (2010) Successful treatment of steroid-resistant nephrotic syndrome associated with *WT1* mutations. Pediatr Nephrol 25: 1285-1289
81. Genovese G, Friedman DJ, Ross MD, Lecordier L, Uzureau P, Freedman BI, Bowden DW, Langefeld CD, Oleksyk TK, Knob ALU, Bernhardy AJ, Hicks PJ, Nelson GW, Vanhollebeke B, Winkler CA, Kopp JB, Pays E, Pollak MR (2010) Association of trypanolytic ApoL1 variants with kidney disease in African Americans. Science 329: 841-845
82. Genovese G, Tonna SJ, Knob AU, Appel GB, Katz A, Bernhardy AJ, Needham AW, Lazarus R, Pollak MR (2010) A risk allele for focal segmental glomerulosclerosis in African Americans is located within a region containing *APOL1* and *MYH9*. Kidney Internat 78: 698-704
83. Ghiggeri GM, Aucella F, Caridi G, Bisceglia L, Ghio L, Gigante M, Perfumo F, Carraro M, Gesualdo L (2006) Posttransplant recurrence of proteinuria in a case of focal segmental glomerulosclerosis associated with WT1 mutation. Am J Transplant 6: 2208-2211
84. Gibson J, Gilbert RD, Bunyan DJ, Angus EM, Fowler DJ, Ennis S (2013) Exome analysis resolves differential diagnosis of familial kidney disease and uncovers a potential confounding variant. Genetics Res Camb 95: 165-173
85. Gigante M, Caridi G, Montemurno E, Soccio M, d’Apolito M, Cerullo G, Aucella F, Schirinzi A, Emma F, Massella L, Messina G, De Palo T, Ranieri E, Ghiggeri GM, Gesualdo L (2011) *TRPC6* mutations in children with steroid-resistant nephrotic syndrome and atypical phenotype. Clin J Am Soc Nephrol 6: 1626-1634 [c, o]
86. Gigante M, Pontrelli P, Montemurno E, Roca L, Aucella F, Penza R, Caridi G, Ranieri E, Ghiggeri GM, Gesualdo L (2009) *CD2AP* mutations are associated with sporadic nephrotic syndrome and focal segmental glomerulosclerosis (FSGS). Nephrol Dial Transplant 24: 1858-1864 [f]
87. Giglio S, Provenzano A, Mazzinghi B, Becherucci F, Giunti L, Sansavini G, Ravaglia F, Roperto RM, Farsetti S, Benetti E, Rotondi M, Murer L, Lazzeri E, Lasagni L, Materassi M, Romagnani P (2015) Heterogeneous genetic alterations in sporadic nephrotic syndrome associate with resistance to immunosuppression. J Am Soc Nephrol 26: 230-236 [o]
88. Gopalakrishnan I, Iskandar SS, Daeihagh P, Divers J, Langefeld CD, Bowden DW, Hicks PJ, Rocco MV, Freedman BI (2011) Coincident idiopathic focal segmental glomerulosclerosis collapsing variant and diabetic nephropathy in an African American homozygous for *MYH9* risk variants. Human Pathol 42: 291-294 [c, f, o]
89. Guaragna MS, Lutaif ACGB, Piveta CSC, Souza ML, de Souza SR, Henriques TB, Maciel-Guerra AT, Belangero VMS, Guerra-Junior G, De Mello MP (2015) *NPHS2* mutations account for only 15 % of nephrotic syndrome cases. BMC Med Genet 16: 88 [o]
90. Guery B, Choukroun G, Noel LH, Clavel P, Rotig A, Lebon S, Rustin P, Bellane-Chantelot C, Mougenot B, Grunfeld JP, Chauveau D (2003) The spectrum of systemic involvement in adults presenting with renal lesion and mitochondrial *tRNA(Leu)* gene mutation. J Am Soc Nephrol 14: 2099-2108 [r, f, o]
91. Guillausseau PJ, Massin P, Dubois-LaForgue D, Timsit J, Virally M, Gin H, Bertin E, Blickle JF, Bouhanick B, Cahen J, Caillat-Zucman S, Charpentier G, Chedin P, Derrien C, Ducluzeau PH, Grimaldi A, Guerci B, Kaloustian E, Murat A, Olivier F, Paques M, Paquis-Flucklinger V, Porokhov B, Samuel-Lajeunesse J, Vialettes B (2001) Maternally inherited diabetes and deafness: a multicenter study. Ann Intern Med 134: 721-728
92. Hall G, Gbadegesin RA, Lavin P, Wu G, Liu Y, Oh EC, Wang L, Spurney RF, Eckel J, Lindsey T, Homstad A, Malone AF, Phelan PJ, Shaw A, Howell DN, Conlon PJ, Katsanis N, Winn MP (2015) A novel missense mutation of Wilms’ Tumor 1 causes autosomal dominant FSGS. J Am Soc Nephrol 26: 831-843 [r, f]
93. Han KH, Lee HK, Kang HG, Moon KC, Lee JH, Park YS, Ha IS, Ahn HS, Choi Y, Cheong HI (2011) Renal manifestations of patients with MYH9-related disorders. Pediatr Nephrol 26: 549-555 [f, o]
94. Hanssen O, Castermans E, Bovy C, Weekers L, Erpicum P, Dubois B, Bours V, Krzesinski JM, Jouret F (2014) Two novel mutations of the *CLDN16* gene cause familial hypomagnesaemia with hypercalciuria and nephrocalcinosis. Clin Kidney J 7: 282-285 [c, f]
95. Has C, Sparta G, Kiritsi D, Weibel L, Moeller A, Vega-Warner V, Waters A, He Y, Anikster Y, Esser P, Straub BK, Hausser I, Bockenhauer D, Dekel B, Hildebrandt F, Bruckner-Tuderman L, Laube GF (2012) Integrin alpha 3 mutations with kidney, lung, and skin disease. N Engl J Med 366: 1508-1514 [f]
96. Hasselbacher K, Wiggins RC, Matejas V, Hinkes BG, Mucha B, Hoskins BE, Ozaltin F, Nurnberg G, Becker C, Hangan D, Pohl M, Kuwertz-Broking E, Griebel M, Schumacher V, Royer-Pokora B, Bakkaloglu A, Nurnberg P, Zenker M, Hildebrandt F (2006) Recessive missense mutations in *LAMB2* expand the clinical spectrum of *LAMB2*-associated disorders. Kidney Internat 70: 1008-1012 [o]
97. He N, Zahirieh A, Mei Y, Lee B, Senthilnathan S, Wong B, Mucha B, Hildebrandt F, Cole DE, Cattran D, Pei Y (2007) Recessive *NPHS2* (podocin) mutations are rare in adult-onset idiopathic focal segmental glomerulosclerosis. Clin J Am Soc Nephrol 2: 31-37
98. Heeringa SF, Chernin G, Chaki M, Zhou W, Sloan AJ, Ji Z, Xie LX, Salviati L, Hurd TW, Vega-Warner V, Killen PD, Raphael Y, Ashraf S, Ovunc B, Schoeb DS, McLaughlin HM, Airik R, Vlangos CN, Gbadegesin R, Hinkes B, Saisawat P, Trevisson E, Doimo M, Casarin A, Pertegato V, Giorgi G, Prokisch H, Rotig A, Nurnberg G, Becker C, Wang S, Ozaltin F, Topaloglu R, Bakkaloglu A, Bakkaloglu SA, Muller D, Beissert A, Mir S, Berdeli A, Ozen S, Zenker M, Matejas V, Santos-Ocana C, Navas P, Kusakabe T, Kispert A, Akman S, Soliman NA, Krick S, Mundel P, Reiser J, Nurnberg P, Clarke CF, Wiggins RC, Faul C, Hildebrandt F (2011) *COQ6* mutations in human patients produce nephrotic syndrome with sensorineural deafness. J Clin Invest 121: 2013-2024 [r, f, o]
99. Heeringa SF, Moller CC, Du J, Yue L, Hinkes B, Chernin G, Vlangos CN, Hoyer PF, Reiser J, Hildebrandt F (2009) A novel *TRPC6* mutation that causes childhood FSGS. PLoS ONE 4: e7771
100. Henderson JM, Alexander MP, Pollak MR (2009) Patients with *ACTN4* mutations demonstrate distinctive features of glomerular injury. J Am Soc Nephrol 20: 961-968 [r, c]
101. Hermle T, Schneider R, Schapiro D, Braun DA, van der Ven AT, Warejko JK, Daga A, Widmeier E, Nakayama M, Jobst-Schwan T, Majmundar AJ, Ashraf S, Rao J, Finn LS, Tasic V, Hernandez JD, Bagga A, Jalalah SM, El Desoky S, Kari JA, Laricchia KM, Lek M, Rehm HL, MacArthur DG, Mane S, Lifton RP, Shril S, Hildebrandt F (2018) *GAPVD1* and *ANKFY1* mutations implicate RAB5 regulation in nephrotic syndrome. J Am Soc Nephrol 29: 2123-2138 [f]
102. Hinkes BG, Mucha B, Vlangos CN, Gbadegesin R, Liu J, Hasselbacher K, Hangan D, Ozaltin F, Zenker M, Hildebrandt F (2007) Nephrotic syndrome in the first year of life: two thirds of cases are caused by mutations in 4 genes (*NPHS1*, *NPHS2*, *WT1*, and *LAMB2*). Pediatrics 119: 907-919 [o]
103. Hinkes B, Wiggins RC, Gbadegesin R, Vlangos CN, Seelow D, Nurnberg G, Garg P, Verma R, Chaib H, Hoskins BE, Ashraf S, Becker C, Hennies HC, Goyal M, Wharram BL, Schachter AD, Mudumana S, Drummond I, Kerjaschki D, Waldherr R, Dietrich A, Ozaltin F, Bakkaloglu A, Cleper R, Basel-Vanagaite L, Pohl M, Griebel M, Tsygin AN, Soylu A, Muller D, Sorli CS, Bunney TD, Katan M, Liu J, Attanasio M, O’Toole JF, Hasselbacher K, Mucha B, Otto EA, Airik R, Kispert A, Kelley GG, Smrcka AV, Gudermann T, Holzman LB, Nurnberg P, Hildebrandt F (2006) Positional cloning uncovers mutations in *PLCE1* responsible for a nephrotic syndrome variant that may be reversible. Nature Genet 38: 1397-1405 [r, o]
104. Hirano M, Konishi K, Arata N, Iyori M, Saruta T, Kuramochi S, Akizuki M (2002) Renal complications in a patient with A-to-G mutation of mitochondrial DNA at the 3243 position of leucine tRNA. Internal Med 41: 113-118 [f]
105. Hocker B, Knuppel T, Waldherr R, Schaefer F, Weber S, Tonshoff B (2006) Recurrence of proteinuria 10 years post-transplant in *NPHS2*-associated focal segmental glomerulosclerosis after conversion from cyclosporin A to sirolimus. Pediatr Nephrol 21: 1476-1479 [f]
106. Hofstra JM, Laine S, van Kuijk WHM, Schoots J, Baltissen MPA, Hoefsloot LH, Knoers NVAM, Berden JHM, Bindels RJM, van der Vlag J, Hoenderop JGJ, Wetzels JFM, Nijenhuis T (2013) New *TRPC6* gain-of-function mutation in a non-consanguineous Dutch family with late-onset focal segmental glomerulosclerosis. Nephrol Dial Transplant 28: 1830-1838 [c]
107. Hori C, Hiraoka M, Yoshikawa N, Tsuzuki K, Yoshida Y, Yoshioka K, Fujisawa K, Tsukahara H, Ohshima Y, Mayumi M (2001) Significance of ACE genotypes and medical treatments in childhood focal glomerulosclerosis. Nephron 88: 313-319
108. Hotta O, Inoue CN, Miyabayashi S, Furuta T, Takeuchi A, Taguma Y (2001) Clinical and pathologic features of focal segmental glomerulosclerosis with mitochondrial *tRNA^Leu(UUR)^* gene mutation. Kidney Internat 59: 1236-1243 [f]
109. Iijima K, Someya T, Ito S, Nozu K, Nakanishi K, Matsuoka K, Ohashi H, Nagata M, Kamei K, Sasaki S (2012) Focal segmental glomerulosclerosis in patients with complete deletion of one *WT1* allele. Pediatrics 129: e1621-e1625 [f]
110. Inui K, Fukushima H, Tsukamoto M, Taniike M, Midorikawa M, Tanaka J, Nishigaki T, Okada S (1992) Mitochondrial encephalomyopathies with the mutation of the mitochondrial *tRNA^Leu(UUR)^* gene. J Pediat 120: 62-66
111. Ismaili K, Wissing KM, Janssen F, Hall M (2009) Genetic forms of nephrotic syndrome: a single-center experience in Brussels. Pediatr Nephrol 24: 287-294 [o]
112. Izu A, Yanagida H, Sugimoto K, Fujita S, Sakata N, Wada N, Okada M, Takemura T (2011) Pathogenesis of focal segmental glomerular sclerosis in a girl with the partial deletion of chromosome 6p. Tohuku J Exp Med 223: 187-192 [f]
113. Jain V, Feehally J, Jones G, Robertson L, Nair D, Vasudevan P (2014) Steroid-resistant nephrotic syndrome with mutations in NPHS2 (podocin): report from a three-generation family. Clin Kidney J 7: 303-305 [o]
114. Jansen JJ, Maassen JA, Van der Woude FJ, Lemmink HAJ, Van den Ouweland JMW, Hart LM, Smeets HJM, Bruijn JA, Lemkes HHPJ (1997) Mutation in mitochondrial *tRNA^Leu(UUR)^* gene associated with progressive kidney disease. J Am Soc Nephrol 8: 1118-1124 [o]
115. Jinks RN, Puffenberger EG, Baple E, Harding B, Crino P, Fogo AB, Wenger O, Xin B, Koehler AE, McGlincy MH, Provencher MM, Smith JD, Tran L, Al Turki S, Chioza BA, Cross H, Harlalka GV, Hurles ME, Maroofian R, Heaps AD, Morton MC, Stempak L, Hildebrandt F, Sadowski CE, Zaritsky J, Campellone K, Morton DH, Wang H, Crosby A, Strauss KA (2015) Recessive nephrocerebellar syndrome on the Galloway-Mowat syndrome spectrum is caused by homozygous protein-truncating mutations of *WDR73*. Brain 138: 2173-2190 [r, f]
116. Jungraithmayr TC, Hofer K, Cochat P, Chernin G, Cortina G, Fargue S, Grimm P, Knueppel T, Kowarsch A, Neuhaus T, Pagel P, Pfeiffer KP, Schafer F, Schonermarck U, Seeman T, Toenshoff B, Weber S, Winn MP, Zschocke J, Zimmerhackl LB (2011) Screening for *NPHS2* mutations may help predict FSGS recurrence after transplantation. J Am Soc Nephrol 22: 579-585
117. Kaltenis P, Schumacher V, Jankauskiene A, Laurinavicius A, Royer-Pokora B (2004) Slow progressive FSGS associated with an F392L *WT1* mutation. Pediatr Nephrol 19: 353-356 [o]
118. Kambham N, Tanji N, Seigle RL, Markowitz GS, Pulkkinen L, Uitto J, D'Agati VD (2000) Congenital focal segmental glomerulosclerosis associated with beta 4 integrin mutation and epidermolysis bullosa. Am J Kidney Dis 36: 190-196 [f, o]
119. Kaneko K, Hasui M, Hata A, Hata D, Nozu K (2010) Focal segmental glomerulosclerosis in a boy with Dent-2 disease. Pediatr Nephrol 25: 781-782 [f]
120. Kao WHL, Klag MJ, Meoni LA, Reich D, Berthier-Schaad Y, Man Li M, Coresh J, Patterson N, Tandon A, Powe NR, Fink NE, Sadler JH, Weir MR, Abboud HE, Adler SG, Divers J, Iyengar SK, Freedman BI, Kimmel PL, Knowler WC, Kohn OF, Kramp K, Leehey DJ, Nicholas SB, Pahl MV, Schelling JR, Sedor JR, Thornley-Brown D, Winkler CA, Smith MW, Parekh RS (2008) MYH9 is associated with nondiabetic end-stage renal disease in African Americans. Nature Genet 40: 1185-1192 [o]
121. Kaplan JM, Kim SH, North KN, Rennke H, Correia LA, Tong HQ, Mathis BJ, Rodriguez-Perez JC, Allen PG, Beggs AH, Pollak MR (2000) Mutations in *ACTN4*, encoding alpha-actinin-4, cause familial focal segmental glomerulosclerosis. Nature Genet 24: 251-256 [f]
122. Kari JA, Montini G, Bockenhauer D, Brennan E, Rees L, Trompeter RS, Tullus K, van’t Hoff W, Waters A, Ashton E, Lench N, Sebire NJ, Marks SD (2014) Clinico-pathological correlations of congenital and infantile nephrotic syndrome over twenty years. Pediatr Nephrol 29: 2173-2180 [r, o]
123. Karle SM, Uetz B, Ronner V, Glaeser L, Hildebrandt F, Fuchshuber A (2002) Novel mutations in *NPHS2* detected in both familial and sporadic steroid-resistant nephrotic syndrome. J Am Soc Nephrol 13: 388-393 [o]
124. Kerti A, Csohány R, Wagner L, Jávorszky E, Maka E, Tory K (2013) *NPHS2* homozygous p.R229Q variant: potential modifier instead of causal effect in focal segmental glomerulosclerosis. Pediatr Nephrol 28: 2061-2064
125. Kikuchi H, Takata A, Akasaka Y, Fukuzawa R, Yoneyama H,Kurosawa Y, Honda M, Kamiyama Y, Hata J (1998) Do intronic mutations affecting splicing of *WT1* exon 9 cause Frasier syndrome? J Med Genet 35: 45-48 [o]
126. Kim JM, Wu H, Green G, Winkler CA, Kopp JB, Miner JH, Unanue ER, Shaw AS (2003) CD2-associated protein haploinsufficiency is linked to glomerular disease susceptibility. Science 300: 1298-1300
127. Klamt B, Koziell A, Poulat F, Wieacker P, Scambler P, Berta P, Gessler M (1998) Frasier syndrome is caused by defective alternative splicing of *WT1* leading to an altered ratio of WT1 +/–KTS splice isoforms. Hum Molec Genet 7: 709-714 [o]
128. Kobayashi A, Goto Y, Nagata M, Yamaguchi Y (2010) Granular swollen epithelial cells: a histologic and diagnostic marker for mitochondrial nephropathy. Am J Surg Pathol 34: 262-270
129. Kohler B, Biebermann H, Friedsam V, Gellermann J, Maier RF, Pohl M, Wieacker P, Hiort O, Gruters A, Krude H (2011) Analysis of the Wilms’ tumor suppressor gene (*WT1*) in patients 46,XY disorders of sex development. J Clin Endocrinol Metab 96: E1131-E1136 [o]
130. Kopp JB, Nelson GW, Sampath K, Johnson RC, Genovese G, An P, Friedman D, Briggs W, Dart R, Korbet S, Mokrzycki MH, Kimmel PL, Limou S, Ahuja TS, Berns JS, Fryc J, Simon EE, Smith MC, Trachtman H, Michel DM, Schelling JR, Vlahov D, Pollak M, Winkler CA (2011) *APOL1* genetic variants in focal segmental glomerulosclerosis and HIV-associated nephropathy. J Am Soc Nephrol 22: 2129-2137 [c, o]
131. Kopp JB, Smith MW, Nelson GW, Johnson RC, Freedman BI, Bowden DW, Oleksyk T, McKenzie LM, Kajiyama H, Ahuja TS, Berns JS, Briggs W, Cho ME, Dart RA, Kimmel PL, Korbet SM, Michel DM, Mokrzycki MH, Schelling JR, Simon E, Trachtman H, Vlahov D, Winkler CA (2008) *MYH9* is a major-effect risk gene for focal segmental glomerulosclerosis. Nature Genet 40: 1175-1184 [c, o]
132. Korkmaz E, Lipska-Zietkiewicz BS, Boyer O, Gribouval O, Fourrage C, Tabatabaei M, Schnaidt S, Gucer S, Kaymaz F, Arici M, Dinckan A, Mir S, Bayazit AK, Emre S, Balat A, Rees L, Shroff R, Bergmann C, Mourani C, Antignac C, Ozaltin F, Schaefer FS (2016) ADCK4-associated glomerulopathy causes adolescence-onset FSGS. J Am Soc Nephrol 27: 63-68 [c, o]
133. Koziell A, Grech V, Hussain S, Lee G, Lenkkeri U, Tryggvason K, Scambler P (2002) Genotype/phenotype correlations of *NPHS1* and *NPHS2* mutations in nephrotic syndrome advocate a functional inter-relationship in glomerular filtration. Hum Molec Genet 11: 379-388 [r, o]
134. Kurogouchi F, Oguchi T, Mawatari E, Yamaura S, Hora K, Takei M, Sekijima Y, Ikeda S, Kiyosawa K (1998) A case of mitochondrial cytopathy with a typical point mutation for MELAS, presenting with severe focal-segmental glomerulosclerosis as main clinical manifestation. Am J Nephrol 18: 551-556 [f]
135. Laurin LP, Lu M, Mott AK, Blyth ER, Poulton CJ, Weck KE (2014) Podocyte-associated gene mutation screening in a heterogeneous cohort of patients with sporadic focal segmental glomerulosclerosis. Nephrol Dial Transplant 29: 2062-2069 [c]
136. Lee DY, Kim W, Kang SK, Koh GY, Park SK (1997) Angiotensin-converting enzyme gene polymorphism in patients with minimal-change nephrotic syndrome and focal segmental glomerulosclerosis. Nephron 77: 471-473 [o]
137. Lee HK, Han KH, Jung YH, Kang HG, Moon KC, Ha IS, Choi Y, Cheong HI (2011) Variable renal phenotype in a family with an *INF2* mutation. Pediatr Nephrol 26: 73-76 [f]
138. Lee JH, Han KH, Lee HK, Kang HG, Moon KC, Shin JI, Hahn H, Park YS, Pai KS, Cho BS, Kim SY, Lee SJ, Ha IS, Choi Y, Cheong HI (2011) Genetic basis of congenital and infantile nephrotic syndromes. Am J Kidney Dis. 58: 1042-1043 [o]
139. Lehnhardt A, Karnatz C, Ahlenstiel-Grunow T, Benz K, Benz MR, Budde K, Buscher AK, Fehr T, Feldkotter M, Graf N, Hocker B, Jungraithmayr T, Klaus G, Koehler B, Konrad M, Kranz B, Montoya CR, Muller D, Neuhaus TJ, Oh J, Pape L, Pohl M, Royer-Pokora B, Querfeld U, Schneppenheim R, Staude H, Sparta G, Timmermann K, Wilkening F, Wygoda S, Bergmann C, Kemper MJ (2015) Clinical and molecular characterization of patients with heterozygous mutations in Wilms Tumor Suppressor Gene 1. Clin J Am Soc Nephrol 10: 825-831 [o]
140. Li J, Ding J, Zhao D, Yu Z, Fan Q, Chen Y, Zhang H, Zhong X, Huang J, Yao Y, Xiao H (2010) *WT1* gene mutations in Chinese children with early onset nephrotic syndrome. Pediatr Res 68: 155-158 [o]
141. Liakopoulos V, Huerta A, Cohen S, Pollak MR, Sirota RA, Superdock K, Appel GB (2011) Familial collapsing focal segmental glomerulosclerosis. Clin Nephrol 75: 362-368 [r, c]
142. Lipska BS, Iatropoulos P, Maranta R, Caridi G, Ozaltin F, Anarat A, Balat A, Gellermann J, Trautmann A, Erdogan O, Saeed B, Emre S, Bogdanovic R, Azocar M, Balasz-Chmielewska I, Benetti E, Caliskan S, Mir S, Melk A, Ertan P, Baskin E, Jardim H, Davitaia T, Wasilewska A, Drozdz D, Szczepanska M, Jankauskiene A, Higuita LMS, Ardissino G, Ozkaya O, Kuzma-Mroczkowska E, Soylemezoglu O, Ranchin B, Medynska A, Tkaczyk M, Peco-Antic A, Akil I, Jarmolinski T, Firszt-Adamczyk A, Dusek J, Simonetti GD, Gok F, Gheissari A, Emma F, 43, Krmar RT, Fischbach M, Printza P, Simkova E, Mele C, Ghiggeri GM, Schaefer F (2013) Genetic screening in adolescents with steroid resistant nephrotic syndrome. Kidney Internat 206-213 [o]
143. Lipska BS, Ranchin B, Iatropoulos P, Gellermann J, Melk A, Ozaltin F, Caridi G, Seeman T, Tory K, Jankauskiene A, Zurowska A, Szczepanska M, Wasilewska A, Harambat J, Trautmann A, Peco-Antic A, Borzecka H, Moczulska A, Saeed B, Bogdanovic R, Kalyoncu M, Simkova E, Erdogan O, Vrljicak K, Teixeira A, Azocar M, Schaefer F (2014) Genotype-phenotype associations in WT1 glomerulopathy. Kidney Internat 85: 1169-1178 [o]
144. Longo I, Porcedda P, Mari F, Giachino D, Meloni I, Deplano C, Brusco A, Bosio M, Massella L, Lavoratti G, Roccatello D, Frasca G, Mazzucco G, Muda AO, Conti M, Fasciolo F, Arrondel C, Heidet L, Renieri A, De Marchi M (2002) *COL4A3/COL4A4* mutations: from familial hematuria to autosomal-dominant or recessive Alport syndrome. Kidney Internat 61: 1947-1956 [o]
145. [Lovric](http://cjasn.asnjournals.org/search?author1=Svjetlana+Lovric&sortspec=date&submit=Submit) S, [Fang](http://cjasn.asnjournals.org/search?author1=Humphrey+Fang&sortspec=date&submit=Submit) H, [Vega-Warner](http://cjasn.asnjournals.org/search?author1=Virginia+Vega-Warner&sortspec=date&submit=Submit) V, Sadowski CE, Gee HY, Halbritter J, Ashraf S, Saisawat P, Soliman NA, Kari JA, Otto EA, Hildebrandt F (2014) Rapid detection of monogenic causes of childhood-onset steroid-resistant nephrotic syndrome. Clin J Am Soc Nephrol 9: 1109-1116 [o]
146. Lovric S, Goncalves S, Gee HY, Oskouian B, Srinivas H, Choi W, Shril S, Ashraf S, Tan W, Rao J, Airik M, Schapiro D, Braun DA, Sadowski CE, Widmeier E, Jobst-Schwan T, Schmidt JM, Girik V, Capitani G, Suh JH, Lachaussee N, Arrondel C, Patat J, Gribouval O, Furlano M, Boyer O, Schmitt A, Vuiblet V, Hashmi S, Wilcken R, Bernier FP, Innes AM, Parboosingh JS, Lamont RE, Midgley JP, Wright N, Majewski J, Zenker M, Schaefer F, Kuss N, Greil J, Giese T, Schwarz K, Catheline V, Schanze D, Franke I, Sznajer Y, Truant AS, Adams B, Desir J, Biemann R, Pei Y, Ars E, Lloberas N, Madrid A, Dharnidharka VR, Connolly AM, Willing MC, Cooper MA, Lifton RP, Simons M, Riezman H, Antignac C, Saba JD, Hildebrandt F (2017) Mutations in sphingosine-1-phosphate lyase cause nephrosis with ichthyosis and adrenal insufficiency. J Clin Invest 127: 912-928 [f, o]
147. Lowik MM, Groenen PJTA, Pronk I, Lilien MR, Goldschmeding R, Dijkman HB, Levtchenko EN, Monnens LA, van den Heuvel LP (2007) Focal segmental glomerulosclerosis in a patient homozygous for a *CD2AP* mutation. Kidney Internat 72: 1198-1203 [c, f]
148. Lowik MM, Hol FA, Steenbergen EJ, Wetzels JFM, van den Heuvel LPWJ (2005) Mitochondrial *tRNA^Leu(UUR)^* mutation in a patient with steroid-resistant nephrotic syndrome and focal segmental glomerulosclerosis. Nephrol Dial Transplant 20: 336-341 [f, o]
149. Lowik MM, Levtchenko EN, Monnens LA, Van Den Heuvel LP (2003) *WT-1* and *NPHS2* mutation analysis in patients with non-familial steroid resistant focal-segmental glomerulosclerosis. Clin Nephrol 59: 143-146
150. Lowik M, Levtchenko E, Westra D, Groenen P, Steenbergen E, Weening J, Lilien M, Monnens L, van den Heuvel L (2008) Bigenic heterozygosity and the development of steroid-resistant focal segmental glomerulosclerosis. Nephrol Dial Transplant 23: 3146-3151 [c]
151. Lucke T, Billing H, Sloan EA, Boerkoel CF, Franke D, Zimmering M, Ehrich JHH, Das AM (2005) Schimke-immuno-osseous dysplasia: new mutation with weak genotype–phenotype correlation in siblings. Am J Med Genet 135A: 202-205
152. Ma J, Pan X, Wang Z, Wang Y, Feng X, Ren H, Zhang W, Chen X, Wang W, Chen N (2011) Twenty-one novel mutations identified in the *COL4A5* gene in Chinese patients with X-linked Alport’s syndrome confirmed by skin biopsy. Nephrol Dial Transplant 26: 4003-4010 [o]
153. Maas RJH, Deegens JKJ, van den Brand JAJG, Cornelissen EAM, Wetzels JFM (2013) A retrospective study of focal segmental glomerulosclerosis: clinical criteria can identify patients at high risk for recurrent disease after first renal transplantation. BMC Nephrol 14: 47
154. Machuca E, Benoit G, Nevo F, Tete MJ, Gribouval O, Pawtowski A, Brandstrom P, Loirat C, Niaudet P, Gubler MC, Antignac C (2010) Genotype-phenotype correlations in non-Finnish congenital nephrotic syndrome. J Am Soc Nephrol 21: 1209-1217 [o]
155. Machuca E, Hummel A, Nevo F, Dantal J, Martinez F, Al-Sabban E, Baudouin V, Abel L, Grunfeld JP, Antignac C (2009) Clinical and epidemiological assessment of steroid-resistant nephrotic syndrome associated with the *NPHS2* R229Q variant. Kidney Internat 75: 727-735 [o]
156. Mademan I, Deconinck T, Dinopoulos A, Voit T, Schara U, Devriendt K, Meijers B, Lerut E, De Jonghe P, Baets J (2013) De novo *INF2* mutations expand the genetic spectrum of hereditary neuropathy with glomerulopathy. Neurology 81: 1953-1958 [c, f]
157. Madhavan SM, O’Toole JF, Konieczkowski M, Ganesan S, Bruggeman LA, Sedor JR (2011) APOL1 localization in normal kidney and nondiabetic kidney disease. J Am Soc Nephrol 22: 2119-2128 [r, f, o]
158. Mallett AJ, McCarthy HJ, Ho G, Holman K, Farnsworth E, Patel C, Fletcher JT, Mallawaarachchi A, Quinlan C, Bennetts B, Alexander SI (2017) Massively parallel sequencing and targeted exomes in familial kidney disease can diagnose underlying genetic disorders. Kidney Internat 92: 1493-1506 [o]
159. Malone AF, Phelan PJ, Hall G, Cetincelik U, Homstad A, Alonso AS, Jiang R, Lindsey TB, Wu G, Sparks MA, Smith SR, Webb NJA, Kalra PA, Adeyemo AA, Shaw AS, Conlon PJ, Jennette JC, Howell DN, Winn MP, Gbadegesin RA (2014) Rare hereditary *COL4A3/COL4A4* variants may be mistaken for familial focal segmental glomerulosclerosis. Kidney Internat 86: 1253-1259 [r, f]
160. Mao J, Zhang Y, Du L, Dai Y, Gu W, Liu A, Shang S, Liang L (2007) *NPHS1* and *NPHS2* gene mutations in Chinese children with sporadic nephrotic syndrome. Pediatr Res 61: 117-122 [o]
161. Matejas V, Al-Gazali L, Amirlak I, Zenker M (2006) A syndrome comprising childhood-onset glomerular kidney disease and ocular abnormalities with progressive loss of vision is caused by mutated *LAMB2*. Nephrol Dial Transplant 21: 3283-3286 [r]
162. Matejas V, Hinkes B, Alkandari F, Al-Gazali L, Annexstad E, Aytac MB, Barrow M, Blahova K, Bockenhauer D, Cheong HI, Maruniak-Chudek I, Cochat P, Dotsch J, Gajjar P, Hennekam RC, Janssen F, Kagan M, Kariminejad A, Kemper MJ, Koenig J, Kogan J, Kroes HY, Kuwertz-Broking E, Lewanda AF, Medeira A, Muscheites J, Niaudet P, Pierson M, Saggar A, Seaver L, Suri M, Tsygin A, Wuhl E, Zurowska A, Uebe S, Hildebrandt F, Antignac C, Zenker M (2010) Mutations in the human laminin beta2 (*LAMB2*) gene and the associated phenotypic spectrum. Hum Mutat 31: 992-1002 [o]
163. Mbarek IB, Abroug S, Omezzine A, Pawtowski A, Gubler MC, Bouslama A, Harbi A, Antignac C (2011) Novel mutations in steroid-resistant nephrotic syndrome diagnosed in Tunisian children. Pediatr Nephrol 26: 241-249 [r]
164. McKenzie LM, Hendrickson SL, Briggs WA, Dart RA, Korbet SM, Mokrzycki MH, Kimmel PL, Ahuja TS, Berns JS, Simon EE, Smith MC, Trachtman H, Michel DM, Schelling JR, Cho M, Zhou YC, Binns-Roemer E, Kirk GD, Kopp JB, Winkler CA (2007) *NPHS2* variation in sporadic focal segmental glomerulosclerosis. J Am Soc Nephrol 18: 2987-2995 [c]
165. Mele C, Iatropoulos P, Donadelli R, Calabria A, Maranta R, Cassis P, Buelli S, Tomasoni S, Piras R, Krendel M, Bettoni S, Morigi M, Delledonne M, Pecoraro C, Abbate I, Capobianchi MR, Hildebrandt F, Otto E, Schaefer F, Macciardi F, Ozaltin F, Emre S, Ibsirlioglu T, Benigni A, Remuzzi G, Noris M (2011) *MYO1E* mutations and childhood familial focal segmental glomerulosclerosis. N Engl J Med 365: 295-306 [r, c]
166. Mestrallet G, Bertholet-Thomas A, Ranchin B, Bouvier R, Frappaz D, Cochat P (2011) Recurrence of a dysgerminoma in Frasier syndrome. PediatrTransplant 15: e53–e55
167. Mir S, Yavascan O, Berdeli A, Sozeri B (2012) *TRPC6* gene variants in Turkish children with steroid-resistant nephrotic syndrome. Nephrol Dial Transplant 27: 205-209 [o]
168. Mistry K, Ireland JHE, Ng RCK, Henderson JM, Pollak MR (2007) Novel mutations in *NPHP4* in a consanguineous family with histological findings of focal segmental glomerulosclerosis. Am J Kidney Dis 50: 855-864 [f]
169. Miyake N, Tsukaguchi H, Koshimizu E, Shono A, Matsunaga S, Shiina M, Mimura Y, Imamura S, Hirose T, Okudela K, Nozu K, Akioka Y, Hattori M, Yoshikawa N, Kitamura A, Cheong HI, Kagami S, Yamashita M, Fujita A, Miyatake S, Tsurusaki Y, Nakashima M, Saitsu H, Ohashi K, Imamoto N, Ryo A, Ogata K, Iijima K, Matsumoto N (2015) Biallelic mutations in nuclear pore complex subunit *NUP107* cause early-childhood-onset steroid-resistant nephrotic syndrome. Am J Hum Genet 97: 555-566 [c, f, o]
170. Monteiro EJ, Pereira AC, Pereira AB, Krieger JE, Mastroianni-Kirsztajn G (2006) [*NPHS2* mutations in adult patients with primary focal segmental glomerulosclerosis.](http://ovidsp.tx.ovid.com/sp-3.3.1a/ovidweb.cgi?&S=MDHOFPOFIJDDBFNMNCCLGCLBNLAAAA00&Complete+Reference=S.sh.18%7c1%7c1) J Nephrol 19: 366-371
171. Montini G, Malaventura C, Salviati L (2008) Early coenzyme Q10 supplementation in primary coenzyme Q10 deficiency. New Engl J Med 358: 2849-2850
172. Moulonguet Doleris L, Hill GS, Chedin P, Nochy D, Bellanne-Chantelot C, Hanslik T, Bedrossian J, Caillat-Zucman S, Cahen-Varsaux J, Bariety J (2000) Focal segmental glomerulosclerosis associated with mitochondrial cytopathy. Kidney Internat 58: 1851-1858 [f]
173. Mucha B, Ozaltin F, Hinkes BG, Hasselbacher K, Ruf RG, Schultheiss M, Hangan D, Hoskins BE, Schulze Everding A, Bogdanovic R, Seeman T, Hoppe B, Hildebrandt F (2006) Mutations in the Wilms’ Tumor 1 gene cause isolated steroid resistant nephrotic syndrome and occur in exons 8 and 9. Pediatr Res 59: 325-331 [o]
174. Munkert A, Helmchen U, Kemper MJ, Bubenheim M, Stahl RAK, Harendza S (2009) Characterization of the transcriptional regulation of the human *MT1-MMP* gene and association of risk reduction for focal-segmental glomerulosclerosis with two functional promoter SNPs. Nephrol Dial Transplant 24: 735-742 [o]
175. Nakamura S, Yoshinari M, Doi Y, Yoshizumi H, Katafuchi R, Yokomizo Y, Nishiyama K, Wakisaka M, Fujishima M (1999) Renal complications in patients with diabetes mellitus associated with an A to G mutation of mitochondrial DNA at the 3243 position of leucine tRNA. Diabetes Res Clin Pract 44: 183-189 [f, o]
176. Nelson GW, Freedman BI, Bowden DW, Langefeld CD, An P, Hicks PJ, Bostrom MA, Johnson RC, Kopp JB, Winkler CA (2010) Dense mapping of *MYH9* localizes the strongest kidney disease associations to the region of introns 13 to 15. Hum Molec Genet 19: 1805-1815 [c, o]
177. Ng DK, Robertson CC, Woroniecki RP, Limou S, Gillies CE, Reidy KJ, Winkler CA, Hingorani S, Gibson KL, Hjorten R, Sethna CB, Kopp JB, Moxey-Mims M, Furth SL, Warady BA, Kretzler M, Sedor JR, Kaskel FJ, Sampson MG (2017) APOL1-associated glomerular disease among African-American children: a collaboration of the Chronic Kidney Disease in Children (CKiD) and Nephrotic Syndrome Study Network (NEPTUNE) cohorts. Nephrol Dial Transplant 32: 983-990
178. Nicolaou N, Coert Margadant C, Kevelam SH, Lilien MR, Oosterveld MJS, Kreft M, van Eerde AM, Pfundt R, Terhal PA, van der Zwaag B, Nikkels PGJ, Sachs N, Goldschmeding R, Knoers NVAM, Renkema KY, Sonnenberg A (2012) Gain of glycosylation in integrin α3 causes lung disease and nephrotic syndrome. J Clin Invest 122: 4375-4387 [f, o]
179. Ogino D, Hashimoto T, Hattori M, Sugawara N, Akioka Y, Tamiya G, Makino S, Toyota K, Mitsui T, Hayasaka K (2016) Analysis of the genes responsible for steroid-resistant nephrotic syndrome and/or focal segmental glomerulosclerosis in Japanese patients by whole-exome sequencing analysis. J Hum Genet 61: 137-141
180. Oktem F, Sirin A, Bilge I, Emre S, Agachan B, Ispir T (2004) *ACE* I/D gene polymorphism in primary FSGS and steroid-sensitive nephrotic syndrome. Pediatr Nephrol 19: 384-389 [o]
181. Oleggini R, Bertelli R, Di Donato A, Di Duca M, Caridi G, Sanna-Cherchi S, Scolari F, Murer L, Allegri L, Coppo R, Emma F, Camussi G, Perfumo F, Ghiggeri GM (2006) [Rare functional variants of podocin (*NPHS2*) promoter in patients with nephrotic syndrome.](http://ovidsp.tx.ovid.com/sp-3.4.1b/ovidweb.cgi?&S=IAPGFPLBNODDLBOANCCLDCOBDBKMAA00&Complete+Reference=S.sh.16%7c1%7c1) Gene Expression 13:59-66 [o]
182. Orloff MS, Iyengar SK, Winkler CA, Goddard KAB, Dart RA, Ahuja TS, Mokrzycki M, Briggs WA, Korbet SM, Kimmel PL, Simon EE, Trachtman H, Vlahov D, Michel DM, Berns JS, Smith MC, Schelling JR, Sedor JR, Kopp JB (2005) Variants in the Wilms’ tumor gene are associated with focal segmental glomerulosclerosis in the African American population. Physiol Genomics 21: 212-221
183. Ozaltin F, Ibsirlioglu T, Taskiran EZ, Baydar DE, Kaymaz F, Buyukcelik M, Kilic BD, Balat A, Iatropoulos P, Asan E, Akarsu NA, Schaefer F, Yilmaz E, Bakkaloglu A (2011) Disruption of PTPRO causes childhood-onset nephrotic syndrome. Am J Hum Genet 89: 139-147 [f, o]
184. Ozcakar ZB, Cengiz FB, Cakar N, Uncu N, Kara N, Acar B, Yuksel S, Ekim M, Tekin M, Yalcinkaya F (2006) Analysis of *NPHS2* mutations in Turkish steroid-resistant nephrotic syndrome patients. Pediatr Nephrol 21: 1093-1096 [o]
185. Pan X, Yan J, Ren H, Zhang W, Shi H, Yu H, Wang C, Hao C, Chen X, Chen N (2004) Detection of *COL4A5* gene mutations in Chinese patients with Alport’s syndrome. Nephrol Dial Transplant 19: 1123-1128 [o]
186. Papazachariou L, Demosthenous P, Pieri M, Papagregoriou G, Savva I, Stavrou C, Zavros M, Athanasiou Y, Ioannou K, Patsias C, Panagides A, Potamitis C, Demetriou K, Prikis M, Hadjigavriel M, Kkolou M, Loukaidou P, Pastelli A, Michael A, Lazarou A, Arsali M, Damianou L, Goutziamani I, Soloukides A, Yioukas L, Elia A, Zouvani I, Polycarpou P, Pierides A, Voskarides K, Deltas C (2014) Frequency of *COL4A3/COL4A4* mutations amongst families segregating glomerular microscopic hematuria and evidence for activation of the unfolded protein response. Focal and segmental glomerulosclerosis is a frequent development during ageing. PloS ONE 9: e115015 [o]
187. Papeta N, Kiryluk K, Patel A, Sterken R, Kacak N, Snyder HJ, Imus PH, Mhatre AN, Lawani AK, Julian BA, Wyatt RJ, Novak J, Wyatt CM, Ross MJ, Winston JA, Klotman ME, Cohen DJ, Appel GB, D’Agati VD, Klotman PE, Gharavi AG (2011) *APOL1* variants increase risk for FSGS and HIVAN but not IgA nephropathy. J Am Soc Nephrol 22: 1991-1996 [o]
188. Park E, Ahn YH, Kang HG, Miyake N, Tsukaguchi H, Cheong HI (2017) *NUP107* mutations in children with steroid-resistant nephrotic syndrome. Nephrol Dial Transplant 32: 1013-1017
189. Phelan PJ, Hall G, Wigfall D, Foreman J, Nagaraj S, Malone AF, Winn MP, Howell DN, Gbadegesin R (2015) Variability in phenotype induced by the podocin variant R229Q plus a single pathogenic mutation. Clin Kidney J 8: 538-542 [o]
190. Philippe A, Nevo F, Esquivel EL, Reklaityte D, Gribouval O, Tete MJ, Loirat C, Dantal J, Fischbach M, Pouteil-Noble C, Decramer S, Hoehne M, Benzing T, Charbit M, Niaudet P, Antignac C (2008) Nephrin mutations can cause childhood-onset steroid-resistant nephrotic syndrome. J Am Soc Nephrol 19: 1871-1878 [o]
191. Piccoli GB, Bonino LD, Campisi P, Vigotti FN, Ferraresi M, Fassio F, Brocheriou I, Porpiglia F, Restagno G (2012) Chronic kidney disease, severe arterial and arteriolar sclerosis and kidney neoplasia: on the spectrum of kidney involvement in MELAS syndrome. BMC Nephrology 13: 9 [f]
192. Pierides A, Voskarides K, Athanasiou Y, Ioannou K, Damianou L, Arsali M, Zavros M, Pierides M, Vargemezis V, Patsias C, Zouvani I, Elia A, Kyriacou K, Deltas C (2009) Clinico-pathological correlations in 127 patients in 11 large pedigrees, segregating one of three heterozygous mutations in the *COL4A3/COL4A4* genes associated with familial haematuria and significant late progression to proteinuria and chronic kidney disease from focal segmental glomerulosclerosis. Nephrol Dial Transplant 24: 2721-2729 [f, o]
193. Pollak MR, Alexander MP, Henderson JM (2007) A case of familial kidney disease. Clin J Am Soc Nephrol 2: 1367-1374 [f]
194. Prasad R, Hadjidemetriou I, Maharaj A, Meimaridou E, Buonocore F, Saleem M, Hurcombe J, Bierzynska A, Barbagelata E, Bergada I, Cassinelli H, Das U, Krone R, Hacihamdioglu B, Sari E, Yesilkaya E, Storr HL, Clemente M, Fernandez-Cancio M, Camats N, Ram N, Achermann JC, Van Veldhoven JP, Guasti L, Braslavsky D, Guran T, Metherell LA (2017) Sphingosine-1-phosphate lyase mutations cause primary adrenal insufficiency and steroid-resistant nephrotic syndrome. J Clin Invest 127: 942-953
195. Quinzii C, Naini A, Salviati L, Trevisson E, Navas P, DiMauro S, Hirano M (2006) A mutation in para-hydroxybenzoate-polyprenyl transferase (COQ2) causes primary coenzyme Q10 deficiency. Am J Hum Genet 78: 345-349
196. Rankin J, Auer-Grumbach M, Bagg W, Colclough K, Duong NT, Fenton-May J, Hattersley A, Hudson J, Jardine P, Josifova D, Longman C, McWilliam R, Owen K, Walker M, Wehnert M, Ellard S (2008) Extreme phenotypic diversity and nonpenetrance in families with the *LMNA* gene mutation R644C. Am J Med Genet 146A: 1530-1542
197. Reeves-Daniel AM, DePalma JA, Bleyer AJ, Rocco MV, Murea M, Adams PL, Langefeld CD, Bowden DW, Hicks PJ, Stratta RJ, Lin JJ, Kiger DF, Gautreaux MD, Divers J, Freedman BI (2011) The *APOL1* gene and allograft survival after kidney transplantation. Am J Transplant 11: 1025-1030 [c, o]
198. Reeves-Daniel AM, Iskandar SS, Bowden DW, Bostrom MA, Hicks PJ, Comeau ME, Langefeld CD, Freedman BI (2010) Is collapsing C1q nephropathy another MYH9-associated kidney disease? A case report. Am J Kidney Dis 55: e21-e24 [c, f]
199. Reiser J, Polu KR, Moller CC, Kenlan P, Altintas MM, Wei C, Faul C, Herbert S, Villegas I, Avila-Casado C, McGee M, Sugimoto H, Brown D, Kalluri R, Mundel P, Smith PL, Clapham DE, Pollak MR (2005) TRPC6 is a glomerular slit diaphragm-associated channel required for normal renal function. Nature Genet 37: 739-744
200. Rodriguez PQ, Lohkamp B, Celsi G, Mache CJ, Grumbach MA, Wernerson A, Hamajima N, Tryggvason K, Patrakka J (2013) Novel *INF2* mutation p. L77P in a family with glomerulopathy and Charcot-Marie-Tooth neuropathy. Pediatr Nephrol 28: 339-343 [f]
201. Ruf RG, Lichtenberger A, Karle SM, Haas JP, Anacleto FE, Schultheiss M, Zalewski I, Imm A, Ruf EM, Mucha B, Bagga A, Neuhaus T, Fuchshuber A, Bakkaloglu A, Hildebrandt F (2004) Patients with mutations in *NPHS2* (podocin) do not respond to standard steroid treatment of nephrotic syndrome. J Am Soc Nephrol 15: 722-732 [o]
202. Ruf RG, Schulthiess M, Lichtenberger A, Karle SM, Zalewski I, Mucha B, Everding AS, Neuhaus T, Patzer L, Plank C, Haas JP, Ozaltin F, Imm A, Fuchshuber A, Bakkaloglu A, Hildebrandt F (2004) Prevalence of *WT1* mutations in a large cohort of patients with steroid-resistant and steroid-sensitive nephrotic syndrome. Kidney Internat 66: 564-570
203. Sadowski CE, Lovric S, Ashraf S, Pabst WL, Gee HY, Kohl S, Engelmann S, Vega-Warner, Fang H, Halbritter J, Somers MJ, Tan W, Shril S, Fessi I, Lifton RP, Bockenhauer D, El-Desoky S, Kari JA, Zenker M, Kemper MJ, Mueller D, Fathy HM, Soliman NA, Hildebrandt F (2015) A single-gene cause in 29.5% of cases of steroid-resistant nephrotic syndrome. J Am Soc Nephrol 26: 1279-1289 [c, o]
204. Sampson MG, Gillies CE, Robertson CC, Crawford B, Vega-Warner V, Otto EA, Kretzler M, Kang HM (2016) Using population genetics to interrogate the monogenic nephrotic syndrome diagnosis in a case cohort. J Am Soc Nephrol 27: 1970-1983 [o]
205. Sanchez-Ares M, Garcia-Vidal M, Antucho EE, Julio P, Eduardo VM, Lens XM, Garcia-Gonzalez MA (2012) A novel mutation, outside of the candidate region for diagnosis, in the inverted formin 2 gene can cause focal segmental glomerulosclerosis. Kidney Internat 83: 153-159 [f]
206. Sanna-Cherchi S, Burgess KE, Nees SN, Caridi G, Weng PL, Dagnino M, Bodria M, Carrea A, Allegretta MA, Kim HR, Perry BJ, Gigante M, Clark LN, Kisselev S, Cusi D, Gesualdo L, Allegri L, Scolari F, D’Agati V, Shapiro LS, Pecoraro C, Palomero T, Ghiggeri GM, Gharavi AG (2011) Exome sequencing identified *MYO1E* and *NEIL1* as candidate genes for human autosomal recessive steroid-resistant nephrotic syndrome. Kidney Internat 80; 389-396
207. Santin S, Ars E, Rossetti S, Salido E, Silva I, Garcia-Maset R, Gimenez I, Ruiz P, Mendizabal S, Nieto JL, Pena A, Camacho JA, Fraga G, Cobo MA, Bernis C, Ortiz A, de Pablos AL, Sanchez-Moreno A, Pintos G, Mirapeix E, Fernandez-Llama P, Ballarin J, Torra R (2009) *TRPC6* mutational analysis in a large cohort of patients with focal segmental glomerulosclerosis. Nephrol Dial Transplant 24: 3089-3096 [o]
208. Santin S, Bullich G, Tazon-Vega B, Garcia-Maset R, Gimenez I, Silva I, Ruiz P, Ballarin J, Torra R, Ars E (2011) Clinical utility of genetic testing in children and adults with steroid-resistant nephrotic syndrome. Clin J Am Soc Nephrol 6: 1139-1148 [o]
209. Santin S, Garcia-Maset R, Ruiz P, Gimenez I, Zamora I, Pena A, Madrid A, Camacho JA, Fraga G, Sanchez-Moreno A, Cobo MA, Bernis C, Ortiz A, de Pablos AL, Pintos G, Justa ML, Hidalgo-Barquero E, Fernandez-Llama P, Ballarin J, Ars E, Torra R (2009) Nephrin mutations cause childhood- and adult-onset focal segmental glomerulosclerosis. Kidney Internat 76: 1268-1276
210. Santin S, Tazon-Vega B, Silva I, Cobo MA, Gimenez I, Ruiz P, Garcia-Maset R, Ballarin J, Torra R, Ars E (2011) Clinical value of *NPHS2* analysis in early- and adult-onset steroid-resistant nephrotic syndrome. Clin J Am Soc Nephrol 6: 344-354 [o]
211. Sato Y, Tsukaguchi H, Morita H, Higasa K, Tran MTN, Hamada M, Usui T, Morito N, Horita S, Hayashi T, Takagi J, Yamaguchi I, Nguyen HT, Harada M, Inui K, Maruta Y, Inoue Y, Koiwa F, Sato H, Matsuda F, Ayabe S, Mizuno S, Sugiyama F, Takahashi S, Yoshimura A (2018) A mutation in transcription factor MAFB causes focal segmental glomerulosclerosis with Duane retraction syndrome. Kidney Internat 94: 396-407 [f]
212. Scaglia F, Vogel H, Hawkins EP, Vladutiu GD, Liu LL, Wong LJC (2003) Novel homoplasmic mutation in the mitochondrial *tRNA^Tyr^* gene associated with atypical mitochondrial cytopathy presenting with focal segmental glomerulosclerosis. Am J Med Genet 123A: 172-178 [f]
213. Schoeb DS, Chernin G, Heeringa SF, Matejas V, Held S, Vega-Warner V, Bockenhauer D, Vlangos CN, Moorani KN, Neuhaus TJ, Kari JA, MacDonald J, Saisawat P, Ashraf S, Ovunc B, Zenker M, Hildebrandt F (2010) Nineteen novel *NPHS1* mutations in a worldwide cohort of patients with congenital nephrotic syndrome (CNS). Nephrol Dial Transplant 25: 2970-2976 [o]
214. Schramm L, Gal A, Zimmermann J, Netzer KO, MD, Heidbreder E, Lopau K, Groene H, MD, Wanner C (2004) Advanced renal insufficiency in a 34-year-old man with Lowe syndrome. Am J Kidney Dis 43: 538-543 [f]
215. Schultheiss M, Ruf RG, Mucha BE, Wiggins R, Fuchshuber A, Lichtenberger A, Hildebrandt F (2004) No evidence for genotype/phenotype correlation in *NPHS1* and *NPHS2* mutations. Pediatr Nephrol 19: 1340-1348 [o]
216. Schumacher V, Scharer K, Wuhl E, Altrogge H, Bonzel KE, Guschmann M, Neuhaus TJ, Pollastro RM, Kuwertz-Broking E, Bulla M, Tondera AM, Mundel P, Helmchen U, Waldherr R, Weirich A, Royer-Pokora B (1998) Spectrum of early onset nephrotic syndrome associated with *WT1* missense mutations. Kidney Internat 53: 1594-1600 [o]
217. Segawa-Takaeda C, Takeda S, Ieki Y, Takazakura E, Haratake J, Wada T, Yokoyama H (2002) Focal glomerulosclerosis expanding from the glomerular vascular pole in a Japanese male with mitochondrial-DNA mutation. Nephrol Dial Transplant 17: 172-174 [f]
218. Sekine T, Konno M, Sasaki S, Moritani S, Miura T, Wong W, Nishio H, Nishiguchi T, Ohuchi MY, Tsuchiya S, Matsuyama T, Kanegane H, Ida K, Miura K, Harita Y, Hattori M, Horita S, Igarashi T, Saito H, Kunishima S (2010) Patients with Epstein–Fechtner syndromes owing to *MYH9* R702 mutations develop progressive proteinuric renal disease. Kidney Internat 78: 207-214 [f]
219. Sen ES, Dean P, Yarram-Smith L, Bierzynska A, Woodward G, Buxton C, Dennis G, Welsh GI, Williams M, Saleem MA (2017) Clinical genetic testing using a custom-designed steroid-resistant nephrotic syndrome gene panel: analysis and recommendations. J Med Genet 54: 795-804 [o]
220. Sethi S, Fervenza FC, Zhang Y, Smith RJH (2012) Secondary focal and segmental glomerulosclerosis associated with single-nucleotide polymorphisms in the genes encoding complement factor H and C3. Am J Kidney Dis 60:316-321 [f, o]
221. Sharma S, Kabra M, Hari P, Dinda AK, Bagga A (2008) *NPHS2* and *WT1* mutations in Indian children with steroid-resistant nephrotic syndrome. Genomic Med 2: 249-250 [o]
222. Spink C, Stege G, Tenbrock K, Harendza S (2013) The *CTLA-4* +49GG genotype is associated with susceptibility for nephrotic kidney diseases. Nephrol Dial Transplant 28: 2800-2805 [o]
223. Stefanou C, Pieri M, Savva I, Georgiou G, Pierides A, Voskarides K, Deltas C (2015) Co-inheritance of functional podocin variants with heterozygous collagen IV mutations predisposes to renal failure. Nephron 130: 200-212 [o]
224. Tan W, Lovric S, Ashraf S, Rao J, Schapiro D, Airik M, Shril S, Gee HY, Baum M, Daouk G, Ferguson MA, Rodig N, Somers MJG, Stein DR, Vivante A, Warejko JK, Widmeier E, Hildebrandt F (2018) Analysis of 24 genes reveals a monogenic cause in 11.1% of cases with steroid-resistant nephrotic syndrome at a single center. Pediatr Nephrol 33: 305-314
225. Taniike M, Fukushima H, Yanagihara I, Tsukamoto H, Tanaka J, Fujimura H, Nagai T, Sano T, Yamaoka K, Inui K, Okada S (1992) Mitochondrial *tRNA^Ile^* mutation in fatal cardiomyopathy. Biochem Biophys Res Comm 186: 47-53
226. Thong KM, Xu Y, Cook J, Takou A, Wagner B, Kawar B, Ong ACM (2013) Cosegregation of focal segmental glomerulosclerosis in a family with familial partial lipodystrophy due to a mutation in *LMNA*. Nephron Clin Pract 124: 31-37 [c, f]
227. Tonna SJ, Needham A, Polu K, Uscinski A, Appel GB, Falk RJ, Katz A, Al-Waheeb S, Kaplan BS, Jerums G, Savige J, Harmon J, Zhang K, Curhan GC, Pollak MR (2008) *NPHS2* variation in focal and segmental glomerulosclerosis. BMC Nephrology 9: 13
228. Toyota K, Ogino D, Hayashi M, Taki M, Saito K, Abe A, Hashimoto T, Umetsu K, Tsukaguchi H, Hayasaka K (2013) *INF2* mutations in Charcot-Marie-Tooth disease complicated with focal segmental glomerulosclerosis. J Periph Nerv Syst 18: 97-98
229. Trautmann A, Schnaidt S, Lipska-Zietkiewicz BS, Bodria M, Ozaltin F, Emma F, Anarat A, Melk A, Azocar M, Oh J, Saeed B, Gheisari A, Caliskan S, Gellermann J, Higuita LMS, Jankauskiene A, Drozdz D, Mir S, Balat A, Szczepanska M, Paripovic D, Zurowska A, Bogdanovic R, Yilmaz A, Ranchin B, Baskin E, Erdogan O, Remuzzi G, Firszt-Adamczyk A, Kuzma-Mroczkowska E, Litwin M, Murer L, Tkaczyk M, Jardim H, Wasilewska A, Printza N, Fidan K, Simkova E, Borzecka H, Staude H, Hees K, Schaefer F (2017) Long-term outcome of steroid-resistant nephrotic syndrome in children. J Am Soc Nephrol 28: 3055-3065 [o]
230. Tsukaguchi H, Sudhakar A, Le TC, Nguyen T, Yao J, Schwimmer JA, Schachter AD, Poch E, Abreu PF, Appel GB, Pereira AB, Kalluri R, Pollak MR (2002) *NPHS2* mutations in late-onset focal segmental glomerulosclerosis: R229Q is a common disease-associated allele. J Clin Invest 110:1659-1666
231. Vodopiutz J, Seidl R, Prayer D, Khan MI, Mayr JA, Streubel B, Steiss JO, Hahn A, Csaicsich D, Castro C, Assoum M, Muller T, Wieczorek D, Mancini GMS, Sadowski CE, Levy N, Megarbane A, Godbole K, Schanze D, Hildebrandt F, Delague V, Janecke AR, Zenker M (2015) *WDR73* mutations cause infantile neurodegeneration and variable glomerular kidney disease. Hum Mutat 36: 1021-1028
232. Voskarides K, Damianou L, Neocleous V, Zouvani I, Christodoulidou S, Hadjiconstantinou V, Ioannou K, Athanasiou Y, Patsias C, Alexopoulos E, Pierides A, Kyriacou K, Deltas C (2007) *COL4A3/COL4A4* mutations producing focal segmental glomerulosclerosis and renal failure in thin basement membrane nephropathy. J Am Soc Nephrol 18: 3004-3016 [f, o]
233. Wang F, Zhang Y, Mao J, Yu Z, Yi Z, Yu L, Sun J, Wei X, Ding F, Zhang H, Xiao H, Yao Y, Tan W, Lovric S, Ding J, Hildebrandt F (2017) Spectrum of mutations in Chinese children with steroid-resistant nephrotic syndrome. Pediatr Nephrol 32: 1181-1192 [o]
234. Wang YY, Rana K, Tonna S, Lin T, Sin L, Savige J (2004) *COL4A3* mutations and their clinical consequences in thin basement membrane nephropathy. Kidney Internat 65: 786-790
235. Warejko JK, Tan W, Daga A, Schapiro D, Lawson JA, Shril S, Lovric S, Ashraf S, Rao J, Hermle T, Schwan TJ, Widmeier E, Majmundar AJ, Schneider R, Gee HY, Schmidt JM, Vivante A, van der Ven AT, Ityel H, Chen J, Sadowski CE, Kohl S, Pabst WL, Nakayama M, Somers MJG, Rodig NM, Daouk G, Baum M, Stein DR, Ferguson MA, Traum AZ, Soliman NA, Kari JA, El Desoky S, Fathy H, Zenker M, Bakkaloglu SA, Muller D, Noyan A, Ozaltin F, Cadnapaphornchai MA, Hashmi S, Hopcian J, Kopp JB, Benador N, Bockenhauer D, Bogdanovic R, Stajic N, Chernin G, Ettenger R, Fehrenbach H, Kemper M, Munarriz RL, Podracka L, Buscher R, Serdaroglu E, Tasic V, Mane S, Lifton RP, Braun DA, Hildebrandt F (2018) Whole exome sequencing of patients with steroid-resistant nephrotic syndrome. Clin J Am Soc Nephrol 13: 53-62 [o]
236. Weber S, Gribouval O, Esquivel EL, Moriniere V, Tete MJ, Legendre C, Niaudet P, Antignac C (2004) *NPHS2* mutation analysis shows genetic heterogeneity of steroid-resistant nephrotic syndrome and low post-transplant recurrence. Kidney Internat 66: 571-579
237. Weins A, Kenlan P, Herbert S, Le TC, Villegas I, Kaplan BS, Appel GB, Pollak MR (2005) Mutational and biological analysis of alpha-actinin-4 in focal segmental glomerulosclerosis. J Am Soc Nephrol 16: 3694-3701
238. Winn MP, Conlon PJ, Lynn KL, Farrington MK, Creazzo T, Hawkins AF, Daskalakis N, Kwan SY, Ebersviller S, Burchette JL, Pericak-Vance MA, Howell DN, Vance JM, Rosenberg PB (2005) A mutation in the *TRPC6* cation channel causes familial focal segmental glomerulosclerosis. Science 308: 1801-1804 (Series described in Winn MP, Conlon PJ, Lynn KL, Howell DN, Slotterbeck BD, Smith AH, Graham FL, Bembe ML, Quarles LD, Pericak-Vance MA, Vance JM (1999) Linkage of a gene causing familial focal segmental glomerulosclerosis to chromosome 11 and further evidence of genetic heterogeneity. Genomics 58: 113-120) [r, f, o]
239. Wong W, Morris MC, Kara T (2013) Congenital nephrotic syndrome with prolonged renal survival without renal replacement therapy. Pediatr Nephrol 28: 2313-2321 [o]
240. Wu Y, Hu P, Xu H, Yuan J, Yuan L, Xiong W, Deng X, Deng H (2016) A novel heterozygous *COL4A4* missense mutation in a Chinese family with focal segmental glomerulosclerosis. J Cell Mol Med 20: 2328-2332 [f]
241. Wuttke M, Seidl M, Malinoc A, Prischl FC, Kuehn EW, Walz G, Kottgen A (2015) A *COL4A5* mutation with glomerular disease and signs of chronic thrombotic microangiopathy. Clin Kidney J 8: 690-694 [f]
242. Xie J, Hao X, Azeloglu EU, Ren H, Wang Z, Ma J, Liu J, Ma X, Wang W, Pan X, Zhang W, Zhong F, Li Y, Meng G, Kiryluk K, He JC, Gharavi AG, Chen N (2015) Novel mutations in the inverted formin 2 gene of Chinese families contribute to focal segmental glomerulosclerosis. Kidney Internat 88: 593-604 [f, o]
243. Xie J, Wu X, Ren H, Wang W, Wang Z, Pan X, Hao X, Tong J, Ma J, Ye Z, Meng G, Zhu Y, Kiryluk K, Kong X, Hu L, Chen N (2014) *COL4A3* mutations cause focal segmental glomerulosclerosis. J Molec Cell Biol 6: 498-505 [o]
244. Yamagata K, Muro K, Usui J, Hagiwara M, Kai H, Arakawa Y, Shimizu Y, Tomida C, Hirayama K, Kobayashi M, Koyama A (2002) Mitochondrial DNA mutations in focal segmental glomerulosclerosis lesions. J Am Soc Nephrol 13: 1816-1823 [f]
245. Yamazaki H, Nozu K, Narita I, Nagata M, Nozu Y, Fu XJ, Matsuo M, Iijima K, Gejyo F (2009) Atypical phenotype of type I Bartter syndrome accompanied by focal segmental glomerulosclerosis. Pediatr Nephrol 24: 415-418 [c, f]
246. Yap DYH, Tse KC, Chan TM, Kwok A, Lie W (2009) Epstein syndrome presenting as renal failure in young patients. Renal Failure 31: 582-585 [f]
247. Yorifuji T, Kawai M, Momoi T, Sasaki H, Furusho K, Muroi J, Shimizu K, Takahashi Y, Matsumura M, Nambu M, Okuno T (1996) Nephropathy and growth hormone deficiency in a patient with mitochondrial *tRNA^Leu(UUR)^* mutation. J Med Genet 33: 621-622 [o]
248. Yu H, Artomov M, Brähler S, Stander MC, Shamsan G, Sampson MG, White JM, Kretzler M, Miner JH, Jain S, Winkler CA, Mitra RD, Kopp JB, Daly MJ, Shaw AS (2016) A role for genetic susceptibility in sporadic focal segmental glomerulosclerosis. J Clin Invest 126: 1067-1078
249. Yu Z, Ding J, Huang J,Yao Y, Xiao H, Zhang J, Liu J, Yang J (2005) Mutations in *NPHS2* in sporadic steroid-resistant nephrotic syndrome in Chinese children. Nephrol Dial Transplant 20: 902-908
250. Yue Z, Xiong S, Sun L, Huang W, Mo Y, Huang L, Jiang X, Chen S, Hu B, Wang Y (2010) Novel compound mutations of *SMARCAL1* associated with severe Schimke immuno-osseous dysplasia in a Chinese patient. Nephrol Dial Transplant 25: 1697-1702 [f]
251. Zhu B, Chena N, Wang Z, Pana X, Rena H, Zhang W, Wang W (2009) Identification and functional analysis of a novel *TRPC6* mutation associated with late onset familial focal segmental glomerulosclerosis in Chinese patients. Mutation Res 664: 84-90 [f]
252. Zhu C, Zhao F, Zhang W, Wu H, Chen Y, Ding G, Zhang A, Huang S (2013) A familial *WT1* mutation associated with incomplete Denys-Drash syndrome. Eur J Pediatr 172: 1357-1362 [f]
